# Survivin prevents the polycomb repressor complex 2 from methylating histone 3 lysine 27

**DOI:** 10.1016/j.isci.2023.106976

**Published:** 2023-05-29

**Authors:** Maja Jensen, Venkataragavan Chandrasekaran, María-José García-Bonete, Shuxiang Li, Atsarina Larasati Anindya, Karin Andersson, Malin C. Erlandsson, Nina Y. Oparina, Björn M. Burmann, Ulrika Brath, Anna R. Panchenko, Maria Bokarewa I., Gergely Katona

**Affiliations:** 1 Department of Chemistry and Molecular Biology, Faculty of Science, University of Gothenburg, Box 462, 405 30 Gothenburg, Sweden; 2 Department of Rheumatology and Inflammation Research, Institute of Medicine, University of Gothenburg, Box 480, 40530 Gothenburg, Sweden; 3 Department of Medical Biochemistry and Cell Biology, Institute of Biomedicine, University of Gothenburg, Box 440, 405 30 Gothenburg, Sweden; 4 Department of Pathology and Molecular Medicine, School of Medicine, Queen’s University, Kingston, ON K7L 3N6, Canada; 5 Wallenberg Centre for Molecular and Translational Medicine, University of Gothenburg, 405 30 Gothenburg, Sweden; 6 Department of Chemistry and Molecular Biology and the Swedish NMR Centre, University of Gothenburg, 412 96 Gothenburg, Sweden; 7 Rheumatology Clinic, Sahlgrenska University Hospital, Gröna stråket 16, 41346 Gothenburg, Sweden

**Keywords:** Biochemistry, Epigenetics, Cell biology

## Abstract

This study investigates the role of survivin in epigenetic control of gene transcription through interaction with the polycomb repressive complex 2 (PRC2). PRC2 is responsible for silencing gene expression by trimethylating lysine 27 on histone 3. We observed differential expression of PRC2 subunits in CD4^+^ T cells with varying levels of survivin expression, and ChIP-seq results indicated that survivin colocalizes with PRC2 along DNA. Inhibition of survivin resulted in a significant increase in H3K27 trimethylation, implying that survivin prevents PRC2 from functioning. Peptide microarray showed that survivin interacts with peptides from PRC2 subunits, and machine learning revealed that amino acid composition contains relevant information for predicting survivin interaction. NMR and BLI experiments supported the interaction of survivin with PRC2 subunit EZH2. Finally, protein-protein docking revealed that the survivin-EZH2 interaction interface overlaps with catalytic residues of EZH2, potentially inhibiting its H3K27 methylation activity. These findings suggest that survivin inhibits PRC2 function.

## Highlights

• Survivin colocalizes with PRC2 on the DNA and binds PRC2 derived peptides
• Chemical composition of the PRC2 peptides predicts survivin binding
• Modelling shows survivin interaction with catalytic residues of PRC2
• Inhibition of survivin in CD4^+^ T cells increases histone H3K27 tri-methylation

## Graphical abstract

**Figure:**
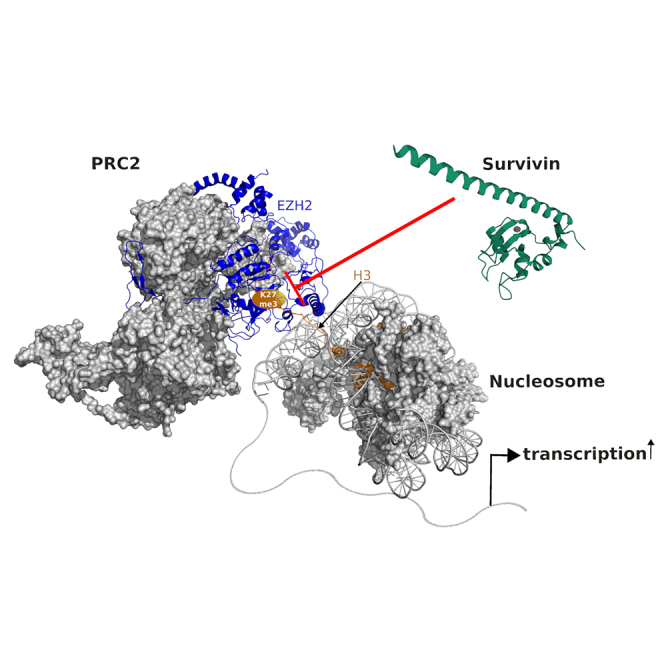

## Introduction

A tight regulation of cellular processes such as apoptosis and cell division is required for the human body to develop and maintain cell homeostasis, and the protein survivin plays a key part in these processes. Survivin is the smallest member of the inhibitors of apoptosis protein (IAP) family, with a length of 142 amino acids and a molecular weight of 16.5 kDa. Survivin, unlike the other members of the IAP family, has a single baculovirus inhibitor of apoptosis repeat connected to the α-helical C-terminus.^1^^,^^2^ The expression level and location of survivin in the cell is highly dependent on the phase of the cell cycle. Survivin, along with the Aurora kinase B, INCENP, and borealin, participates in the chromosomal passenger complex (CPC) during cell division.^3^^,^^4^ Survivin is found in the cytoplasm during the interphase when it is not part of the CPC and prevents apoptosis. Smac/DIABLO is released from the mitochondria during apoptotic signaling and hinders survivin from inhibiting apoptosis. The role of survivin in gene transcription control is a largely unexplored function. A more systematic search for survivin-binding sites in the chromatin is required to support this emerging concept.^5^ In order to map potential association with chromatin, we performed deep sequencing of DNA regions bound by survivin in CD4^+^ T cells.

We found that the precipitated regions were strongly associated with chromatin locations of subunits associated with polycomb repressive complex 2 (PRC2). PRC2 is a histone methyltransferase that methylates Lys-27 on histone 3 (H3K27), hence controlling gene expression by epigenetic modification.^6^ When H3K27 is trimethylated, the genes are maintained in a repressed state, preserving the identity of the cell.^7^ PRC2 is intensively studied because of its role in various types of cancer and its ability to restore pluripotency.^8^^,^^9^^,^^10^ Its core subunit structure is well described and consists of four subunits: enhancer of zeste homolog 2 (EZH2), embryonic ectoderm development (EED), suppressor of zeste 12 (SUZ12), and retinoblastoma-binding protein 46/48 (RBAP48). EZH2 contains the PRC2 enzyme active site, which is responsible for the trimethylation of H3K27. The complex’s smallest component is the seven WD40-repeat protein EED and EED’s aromatic cage consists of four aromatic side chains: Phe-97, Tyr-148, Trp-364, and Tyr-365. This motif binds to already triple-methylated H3K27, enhancing PRC2 activity on other histone subunits.^11^ SUZ12 accumulates the PRC2 subunits, contributing to the complex’s stability and keeping them together.^12^ PRC2 co-factors have been reported as Jumonji and AT-rich interaction-containing domain 2 (JARID2), as well as adipocyte enhancer-binding protein 2 (AEBP2). JARID2 mimics a methylated H3 tail, which activates PRC2. In contrast, AEBP2 mimics an unaltered H3 tail.^12^

EZH2 is the primary mediator of PRC2 function in gene regulation. EZH2 activity is essential for normal cell homeostasis, whereas abnormal expression has been linked to proliferation, cell cycle progression, and oncogenesis.^8^^,^^9^^,^^10^ Recent studies revealed the importance of EZH2 in hematopoetic stem cells, thymopoesis, and lymphopoesis.^13^^,^^14^ Notably, T cell development and function are severely hampered by EZH2 depletion.^15^^,^^16^^,^^17^ This makes it an attractive intervention target in various forms of cancer and autoimmune conditions.^18^^,^^19^^,^^20^

Here, we studied the role of survivin on chromatin of CD4^+^ T cells and identified its interaction with PRC2. We specifically examined the direct interaction of PRC2 with survivin using peptide microarrays, biolayer interferometry (BLI), and nuclear magnetic resonance (NMR) spectroscopy. Machine learning analysis revealed that the interaction between survivin and PRC2-derived peptides is primarily governed by their amino acid/atomic composition rather than the sequence. We used small-angle X-ray scattering (SAXS) and microscale thermophoresis (MST) techniques to investigate the oligomerization state of survivin at a range of concentrations. We performed protein-protein docking modeling to support the feasibility of the proposed interaction, which predicted the survivin interaction with the active site of the EZH2 SET domain, disrupting its methyltransferase activity and abrogating H3K27 trimethylation.

## Results and discussion

### Colocalization of survivin and histone H3K27me3 on the chromatin of primary CD4^+^ T cells

It is known that survivin binds nucleosomes through phosphorylated Thr-3 of histone H3.^21^ Although PRC2 also targets the N-terminal tail of H3, the two actors may not be present at the same time. We explored if H3K27 histone modifications involved in epigenetic regulation colocalize with survivin using ChiP-seq analysis in human primary CD4^+^ T cells. DNA material of CD4^+^ T cells cultured in presence of ConA LPS was precipitated with antibodies against survivin, H3K27me3, and H3K27ac. The immunoprecipitated (IP) DNA was sequenced and mapped to the human genome to identify the DNA regions enriched in the IP material, hereafter termed “peaks”.

**Figure 1:**
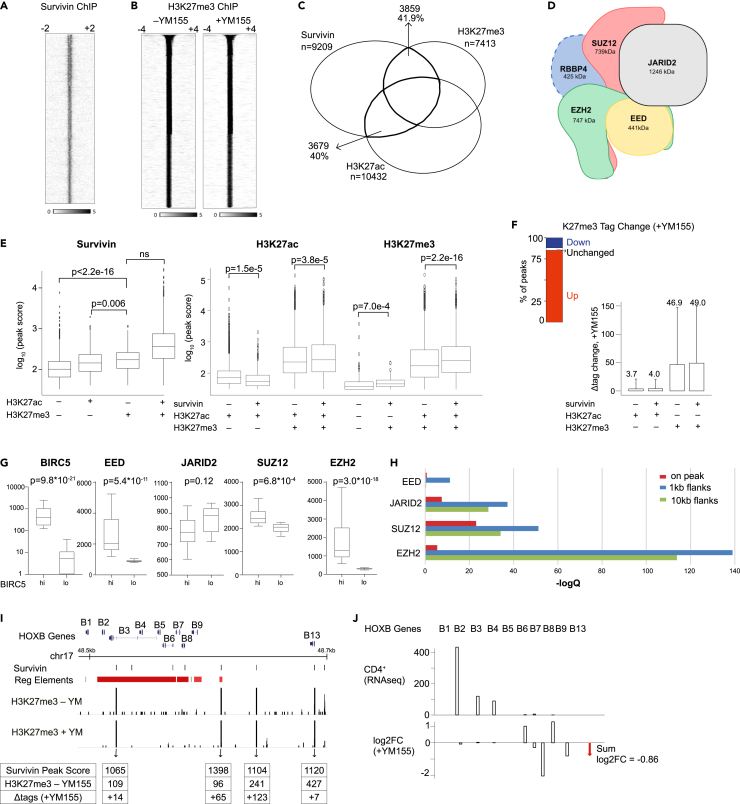
Survivin colocalizes with histone H3K27 marks and controls PRC2 function in human CD4^+^ cells, related to Table S1 (A and B) Heatmap depicts survivin (A) and histone H3K27me3 (B) ChIPseq peaks in human primary CD4^+^ cells, before and after YM155 treatment. (C) Venn diagram of the genomic colocalization between survivin with histone H3K27 ChIPseq peaks in CD4^+^ cells. The peaks overlapping by at least 1 bp were considered colocalized. (D) A cartoon depiction of the PRC2 complex. (E) Peak scores of survivin and histone H3K27 epigenetic marks at colocalization sites. Median, IQR and range indicated. Kolmogorov-Smirnov (KS) test p values are indicated. (F) Proportion change in H3K27me3 peaks after YM155 treatment. The column plot presents the tag count change in H3K27me3 and H3K27ac peaks. Mean and SD indicated. (G) Expression of the PRC2 genes in CD4^+^ cells (n = 24) with high and low survivin/BIRC5, by RNA-seq. Samples were split on the median level of BIRC5 and analyzed using DESeq2. Median with IQR and range, normalized p values indicated. (H) Genomic colocalization of survivin ChIP peaks with the proteins of PRC2 complex. Regions around survivin peaks with 0, 1, and 10 kb flanks were investigated. ChIP-seq data of EZH2, SUZ12, EED, and JARID2 were obtained through the ReMap database. (I) The cluster of HOX-B genes in chromosome 17 is transcriptionally controlled by PRC2. Location of survivin and H3K27me3 peaks in the genome browser is indicated. *Cis*-regulatory elements were retrieved from the GeneHancer database. Increase in H3K27me3 peaks colocalized with survivin after YM155 treatment. (J) Expression of the HOX-B genes in human CD4^+^ cells and the expression change after YM155 treatment, by RNAseq. Red arrow on the right indicates the cumulative reduction in the expression of HOX-B genes after survivin inhibition.

Survivin peaks and H3K27me3 peaks of human primary CD4^+^ T cells obtained in ChIP-seq experiments are shown in Figure 1A and 1B, respectively. Mapping of the survivin and H3K27me3 peaks to the genome revealed that a significant proportion of these peaks colocalized to the same DNA regions (Figure 1C). In addition to a substantial overlap, the colocalization of survivin and histone H3K27 peaks was associated with higher peak scores (Figure 1E). The intersection of the histone modifications was associated with the highest score of survivin peaks, which argues for the affinity of survivin and histone H3K27 marks to those regions in material of the primary CD4^+^ T cells (Figure 1E).

In the next step, we investigated if survivin ChIP-seq peak locations overlap with deposition of the core PRC2 subunits EZH2, EED, SUZ12, and JARID2 (Figure 1D) supporting a potential concerted action toward the H3K27 residue. The integration of survivin peaks with human ChIP-seq mega data for individual PRC2 subunits was available through the ReMap database. It demonstrated a significant degree of colocalization between survivin peaks and all PRC2 subunits (Figure 1H). Specifically, the proteins of the PRC2 complex were identified within the top 10% of the probable survivin partners on the chromatin and were dominated by the association of survivin peaks with the catalytic EZH2 subunit. The predicted association had the strongest power when the colocalization of survivin peaks was analyzed within the 1k base interval of transcription factors in contrast to the direct overlap or the wider 10k base window. This observation suggested that survivin may not directly compete with PRC2 for the same DNA region, but rather acts within the same nucleosome. To pave a functional link between the two systems, we utilized 28 RNA-seq datasets of primary CD4^+^ T cells to study the transcription of the PRC2 complex. Figure 1G shows a strong association of the core PRC2 subunit expression with the expression level of survivin and thus presents an additional piece of evidence for concomitant action of survivin and PRC2.

To further investigate the functional role of survivin in the modification of the histone H3K27 residue, we performed ChIP-seq studies culturing CD4^+^ T cells with and without sepantronium bromide (YM155) present, which specifically inhibits survivin transcription,^22^ but does not change the expression of XIAP, cIAP2, Bcl-XL, Bcl-2, Bad,^22^ p53, cIAP1, or STAT3.^23^ A comparison of H3K27 peaks in YM155-treated cells revealed that there was an increase of H3K27me3 deposition in 85% of the H3K27me3 peaks (Figures 1B and 1F). A substantial increase was observed in tag change of H3K27me3 modification upon survivin inhibition, whereas PRC2-independent H3K27 acetylation remained virtually unaffected (Figure 1F). These results imply that binding of survivin in proximity of a H3K27 residue may prevent PRC2 from methylating H3K27, which is important for epigenetic silencing of sensitive genes.

To search for transcriptional changes in genes with H3K27me3 marks across the genome, we first identified 7413 H3K27me3 peaks that increased the count in YM155-treated CD4^+^ cell cultures by 30%, and then used the GeneHancer database to annotate those peaks to 3715 regulatory elements (RE). Integration of genes connected to the RE with RNA-seq of YM155-treated CD4^+^ cells revealed that 157 of the protein-coding genes significantly changed their mRNA levels (nominal p < 0.01) (Table S1). Most of these genes (100 of 157) had a downregulation of the expression, which agreed with a repressive function of the H3K27me3 epigenetic mark.

The Homeobox (HOX) genes that virtually define the body morphology and tissue-specific developmental path of a cell^24^ are critically dependent on modifications of the H3K27 residue for their transcription and to form focal clusters centered on the H3K27me3 mark.^25^ Importantly, deletion of PRC2 components results in transcriptional changes of those genes in human embryonic fibroblasts.^26^ Thus, we have chosen the cluster of HOX-B genes within chromosome 17 to exemplify the functional effect of survivin on the deposition of histone H3K27me3 in this region. We highlighted survivin and H3K27me3 peaks in CD4^+^ T cells and observed a colocalization of four independent survivin and H3K27me3 peaks, three of which were annotated to the caudal HOX-B genes between HOX-B8 and HOX-B13 (Figure 1I). Inhibition of survivin with YM155 resulted in an increase in the size of H3K27me3 peaks, measured by tag counts (Figure 1I). The tag counts for the middle H3K27me3 peaks showed an increase by 51% and 69%, respectively. Transcriptionally, the increase in deposition of inactive H3K27me3 mark led to a change of the HOX-B genes, which was clearly seen in the caudal HOX-B8, -B9, and -B13 genes controlled by those peaks (Figure 1J). While we cannot rule out the possibility that YM155 (or other survivin suppression approaches) indirectly affects PRC2 activity, these inhibition data should not be viewed in isolation from the precise colocalization of survivin and PRC2 subunits along the DNA and their affinity for H3 tails.

### Interaction between survivin and peptides derived from PRC2 subunits

**Figure 2:**
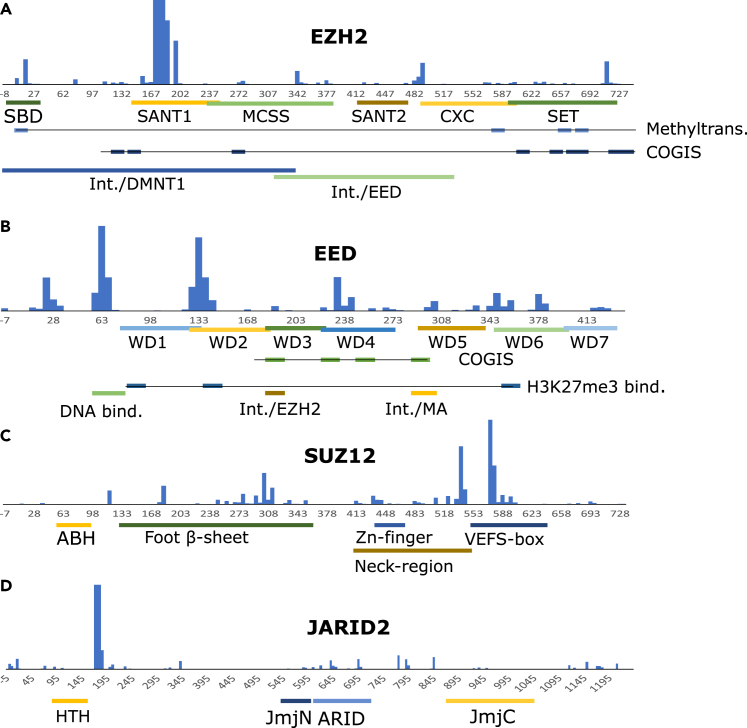
Fluorescence intensity in survivin-peptide microarray, related to Tables S2–S5 and Figure S1 The peptides were derived from (A) EZH2, (B) EED, (C) SUZ12, and (D) JARID2. The residue numbers refer to the first amino acid of peptides. Negative numbers mean filling amino acid residues that are not part of the natural sequence of the subunit. The annotation bars are inclusive, which means that one amino acid residue from the region on a peptide is enough to be marked as part of the region. Because typically three peptides have the same amino acid position, the annotation bars overlap even when the described regions do not.

**Figure 3:**
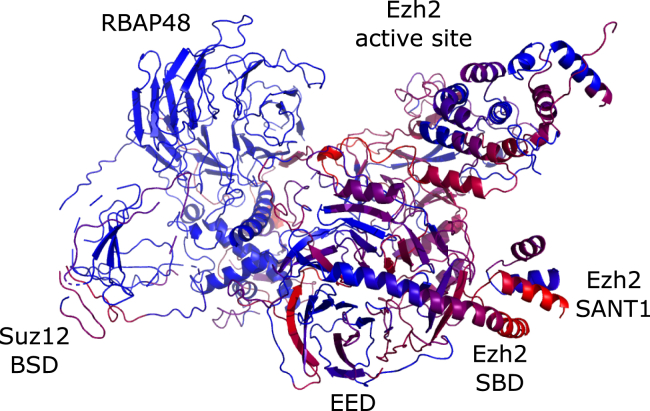
Fluorescence intensity mapped on the 3D structure of the PRC2 core complex The 3D structure is based on PDB ID 6C23.^12^ Blue indicates no interaction or, in the case of subunits RBAP48 and AEBP2, no information about interaction. Progressively brighter red indicates logarithmic magnitude of fluorescence intensity in the peptide microarray experiment. The coloring is inclusive i.e. the residue is associated with the average fluorescence intensity of all the peptides the amino acid residue is present on.

We were looking for potential direct interactions between survivin and short peptide sequences originating from PRC2 subunits and other proteins involved in histone modifications based on the *in vivo* results. Therefore, we created a peptide microarray of overlapping peptides obtained from EZH2, EED, SUZ12, and JARID2 to investigate how survivin interacts with the activity of the PRC2 complex. These subunits form the core of PRC2, and the interaction of survivin with the PRC2 molecular scaffold indicates a potentially disruptive role for survivin in PRC2 function. The fluorescence signal generated by labeled survivin is compared in Figure 2 as a function of position along the polypeptide chain of PRC2 subunits. Figure S1 depicts the raw fluorescence images. Moreover, the microarray fluorescence intensities projected on the three-dimensional structure of the PRC2 core complex are shown in Figure 3. The whole dataset is deposited at the Zenodo data base.

The strongest interaction with EZH2-derived peptides was observed in one continuous region (172–191). The region is located in the SANT1 domain of the enzyme, which is a general binding motif for histone tails and promotes chromatin association.^27^ A strong continuous signal was also observed at peptides in the region 707–736, which included the catalytic residue Tyr-726. Tyr-726 was initially proposed to be the general base for deprotonation of the substrate lysine, but it is more likely that the solvent acts as a general base.^28^^,^^29^ Nevertheless, this is an important part of the active site and when the 728–746 region is missing^30^ or Tyr-736^30^ (alternatively Glu-740^31^) is mutated in humans, it results in a specific phenotype described as Weaver syndrome, a type of overgrowth disease in the affected patients.^30^^,^^32^ Lower level of fluorescence was also observed in peptides covering the 627–667 region. Tyr-641 is also part of the active site region that brings together the SAM cofactor methyl donor and the H3K27 substrate.^33^ The Tyr-641-Phe mutation in EZH2 results in higher H3K27 trimethylation activity.^29^ The adjacent 660–666 region plays an important role for binding pyridone-containing inhibitors of EZH2.^34^ The active site residue Cys-588 is also important for the methyltransferase activity of EZH2 enzyme and peptides containing this residue display moderately high fluorescence (Figure 2A and Table S2). In addition, survivin binding was observed in a continuous region in the N-terminal direction from the SANT1 region where mutations were associated with overgrowth syndrome (Pro-132 & Tyr-133).^35^

The substrate-binding domain (SBD) is located on the N-terminal region of the enzyme, and it is linked to DNA binding. This domain contains Ser-21, which is targeted by phosphorylation. Intriguingly, a mutation at this position affects the methyltransferase activity of EZH2.^36^ This region is surprisingly far from the active site in the PRC2 core structure.^12^ Notably, both the entire N-terminal region (residues 1–36) and peptides containing the residues of the active site display high, continuous fluorescence in the survivin interaction assay. The involvement of survivin may interfere or facilitate the connection between these already distant sites and at the same time, it may influence the DNA-binding properties of EZH2.

In subunit EED, we see repeated (Figure 2B and Table S3) and more evenly distributed regions of high colocalization with survivin including regions 13–37, 58–77, 123–147, and 223–247. The separation of these regions loosely follows the periodicity of the WD repeats, and the regions are concentrated on one side of the doughnut-shaped protein. There is a substantial overlap between survivin-interacting peptides and the 70–79 region of EED. This region is highly basic and contributes to DNA binding and chromatin positioning.^37^ Similarly, a mutation at the conserved site Arg-236 is also known to be involved in the EED-linked Weaver syndrome.^38^^,^^39^ Almost all residues that participate in the binding of the trimethylated lysine are present in the peptides that have high fluorescence signals in the survivin microarray. In addition, H1K26, H3K9, and H3K27 are part of an AXK motif, where the alanine residue of this motif fits into a small pocket on the surface of EED formed by the hydrophobic moieties of Trp-364, Tyr-308, and Cys-324.

In SUZ12 (Figure 2C and Table S4), the highest affinity peptides (533–607) are concentrated in the C-terminal VEFS-box domain, but a continuous region with low binding affinity is present between the residue range 248–367. Peptides from the zinc finger region (448–471) have relatively small, but noticeable fluorescence signals. Mutation Glu-610-Val in SUZ12 is known for association with Weaver syndrome.^38^ This amino acid residue belongs to the VEFS-box region of the protein.

JARID2 contains a sharply defined interacting region between residues 165–199 and several moderately fluorescent regions. (Figure 2D and Table S5) The ARID domain is required to direct the PRC2 complex to its target location on chromatin and this domain is abundant with overlapping peptides interacting with survivin.^40^

### Prediction of survivin-protein interaction governed by amino acid composition

Because of the massive number of peptides (n = 5395) on the microarray, machine learning algorithms were able to characterize the features that promote a peptide to interact with survivin. On the microarray, around 40% of the peptides had fluorescence intensities greater than zero, and approximately 20% of the peptides had fluorescence intensities more than 1000 (Figure S2). Seven peptides with high histidine content were eliminated because they interacted significantly with the anti-His-tag antibody. Thus, the proportions of interacting and non-interacting peptides in our microarray experiment were adequately balanced.

**Figure 4:**
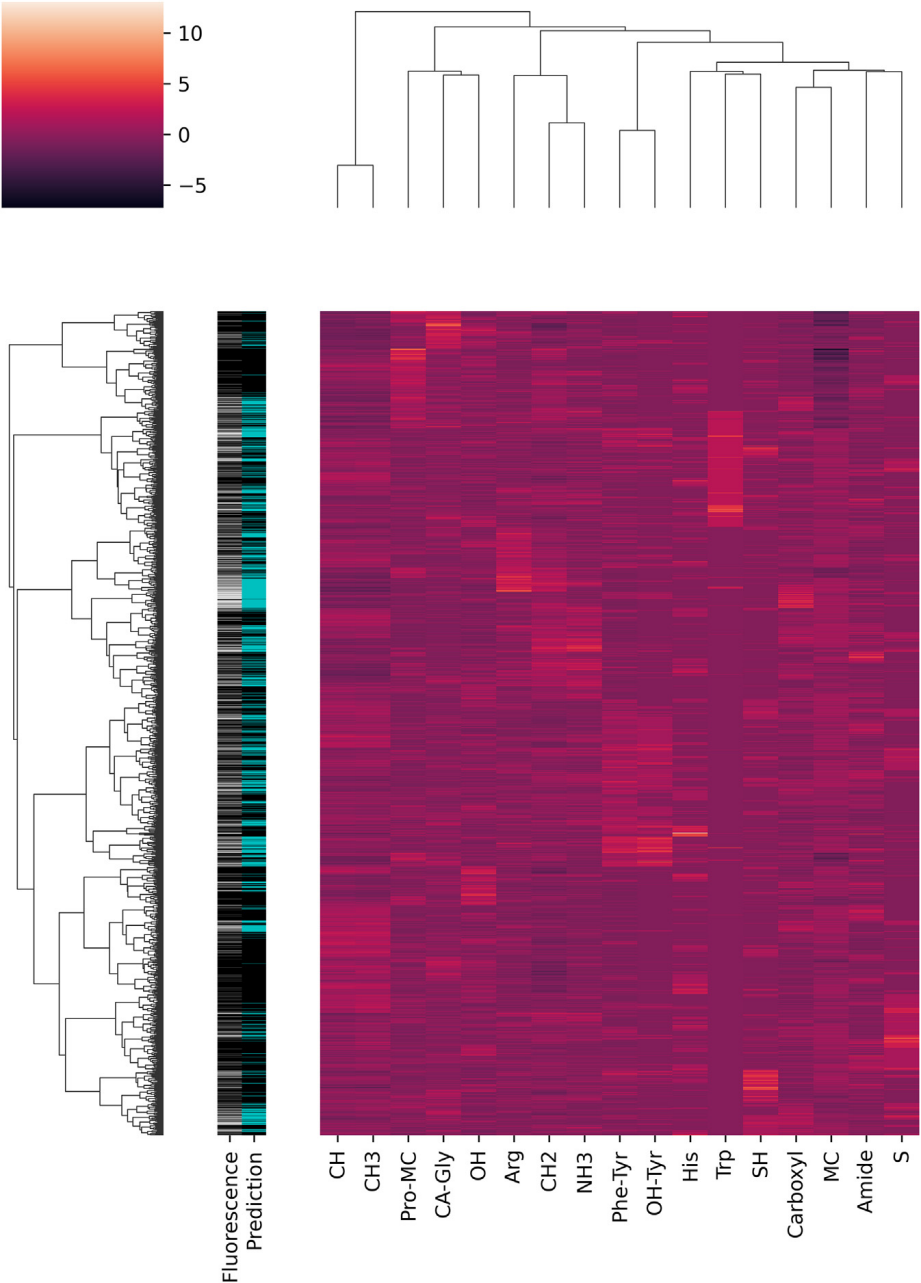
Clustering of peptides based on their atom type abundance, related to Figure S3 The light/dark colors indicate high/low abundance of atom types, respectively. The grayscale bar indicates the logarithm of fluorescence intensity of the peptide in the survivin peptide microarray experiment (*black* zero intensity, *white* highest level of intensity). The prediction bar shows the success of the machine learning prediction using the atom type abundance as features (*black* and *cyan* colors mark predicted non-interacting and interacting peptides, respectively).

Rather than focusing on the sequence of the peptides, the peptides were described based on the abundance of particular atom types in their amino acids (Table S6). The objective of this method was to establish what role the chemical/positional character of atoms plays in biomolecular interactions. The peptides were first clustered using Ward’s method based on atom abundance (Figures 4 and S3). The clustering demonstrated that the similarity of peptides in atom type abundance relates to the fluorescence intensity mediated by survivin interaction. On a larger scale, the accumulation of interacting peptides can be seen, which is linked to specific atom compositions. The dendrogram comprises numerous main branches with a high frequency of interacting peptides, as well as comparably large branches that do not or have a very low frequency of interacting peptides. This does not appear to be a straightforward function of chemical group presence or absence, such as the presence of a large number of carboxyl groups. Simple decision trees, an emphasis on excessively large net charges, or equivalent extremes of hydrophobicity are inadequate for determining whether a peptide is interacting. For a peptide to interact with survivin, a certain combination of atom types must be enhanced/depleted. A magnified section of the cluster (Figure S3) shows that, despite the varying density of interacting peptides in the global dendrogram, the compatibility of a peptide for interaction is highly fine-grained. When interacting with survivin, even peptides with extremely similar compositions can behave differently. As a result, clustering based just on similarity measures may be suboptimal.

To continue the investigation, the peptides were categorized as interacting or non-interacting based on the intensity of the associated fluorescence, and a multilayer perceptron classifier was trained on half of the dataset to detect these two classes based on atom composition features. On the remaining half of the dataset, the prediction strength was assessed. Table 1 depicts the confusion matrix. Furthermore, the predicted interacting peptides are highlighted in cyan in Figures 4 and S3. Although approximately two-thirds of the interacting peptides (precision 0.70, recall 0.65) were correctly recognized, the prediction specificity and sensitivity appeared to be the most successful for the non-interacting peptides (precision 0.80, recall 0.84), providing a solid foundation for detecting non-interacting or repulsive peptides. The predictions replicate the fluorescence signal in Figure 4 remarkably well, and the network frequently correctly predicts the interacting peptides even among extremely similar peptides on a more local scale (Figure S3).

**Table 1:** Confusion matrices of predictions

	Predicted label			
	All peptides		Non-overlapping peptides	
	Non-interacting	Interacting	Non-interacting	Interacting
Correct label				
Non-interacting	1422	276	464	101
Interacting	348	652	119	215
Precision/recall	0.80/0.84	0.70/0.65	0.80/0.82	0.68/0.64

Since the peptides overlap in sequence, the sequence similarity between the test and training set could potentially contaminate the test set. By considering every third peptide only for the test and training set, we eliminated the effect of the overlaps. The relative proportions of elements in the confusion matrix are very similar to relative proportions of elements in the confusion matrix when the classification included every peptide. Therefore, we concluded that the prediction strength did not originate from the training set contaminating the test set. The prediction of interacting peptides did not improve when the interacting peptides with fluorescence intensity below 100 were excluded from the training and testing sets (Table 2).

**Table 2:** Confusion matrices of predictions with weak interaction partners (fluorescence intensity (FI) lower than 100, but larger than 0) excluded from the analysis

	Predicted label			
	All peptides FI > 100 or = 0		Non-overlapping peptides FI > 100 or = 0	
	Non-interacting	Interacting	Non-interacting	Interacting
Correct label				
Non-interacting	1450	282	477	93
Interacting	297	610	105	205
Precision/recall	0.83/0.84	0.68/0.67	0.82/0.84	0.69/0.66

We also assessed the distinctness of peptide features as described by amino acid composition. Only eight peptides were identified with at least one pair with identical amino acid composition, which, once again, cannot explain the robustness of predictions. The near-exclusive uniqueness of peptides is expected from a random model, which assumes uniform probability of the 20 amino acids to occur at any position in the 15 amino acid long peptide. The probability of obtaining a specific peptide sequence is $\frac{1}{20^{15}}=3.1\times 10^{-20}$, but the abundance of amino acids is not specific to a particular sequence. The average probability of obtaining a signature composition in a 15 amino acid peptide can be approximated using the reciprocal combination with repetition ($\frac{15!\left(20-1\right)!}{\left(15+20-1\right)!}=5.4\times 10^{-10}$). Clearly, this will be an extremely asymmetric distribution, with certain combinations, such as 15 identical amino acids, being orders of magnitude less frequent than others. A multinomial distribution with parameters 15 trials with uniform probability of 0.05 for each of the 20 amino acids can describe the entire multivariate distribution. In this microarray design, 10 amino acids were constrained between adjacent peptides, reducing peptide variability even further. Adjacent peptides with identical amino acid content are still rare using the random model ($\frac{5!\left(20-1\right)!}{\left(5+20-1\right)!}=2.4\times 10^{-5}$) in an array of 5395 peptides. Nonetheless, functional protein sequences are not evolved at random, as evidenced by the frequent occurrence of sequences with a high compositional bias (in Table 3 for instance). Non-randomness also explains the appearance of a modest fraction of peptides with identical amino acid compositions, and such repeated occurrences were only present in polypeptide chains from the same protein.

**Table 3:** Interaction strength of the compared peptides as judged from the peptide microarray and the NMR experiments

Subunit	Residues	Peptide sequence	Average FI (%)	Integrated FI	NMR cpc (μM)
EZH2	172–211	VELVNALGQYNDDDDDDDGD DPEEREEKQKDLEDHRDDKE	71	4.28	2.1 × 10^−3^
EED	128–152	SQGEIRLLQSYVDADADENFYTCAW	10	0.31	7.6 × 10^−3^
SUZ12	573–597	PLRPQEMEVDSEDEKDPEWLREKTI	20	0.6	6.6 × 10^−3^
JARID2	170–194	PNSMVYFGSSQDEEEVEEEDDETED	100	3.00	8.5 × 10^−3^

The average fluorescence intensity (FI) is expressed as a percentage of maximum fluorescence intensity units (64193) per peptide. The integrated FI is expressed in units of the maximum FI per peptide. The analyzed microarray peptides contained the amino acid positions of the peptides in this table with as few extra amino acids on the N- and C-termini as possible.

The location of survivin on the peptide microarray was clearly correlated with the amino acid content of the peptide at 1 μg/mL survivin concentration. When evaluating how accurate the predictions were, one should keep in mind that the primary and higher-order structures of the peptide were not at all considered in this analysis. Mislabeling peptides is possible for multiple reasons. The smallest fluorescence signal was chosen as the limit of detection and photon-counting errors may cause weak interactions to be undetected at low fluorescence intensities. Moreover, if the fluorescence intensity of experimental duplicates varied by more than 40%, a peptide was classified as non-interacting even if the lack of reproducibility is ambiguous evidence for the lack of interaction. Nonetheless, we can conclude that the criteria governing peptide interactions do not always require order of amino acids in a sequence. They are most likely basic because they were inferred from a limited training sample set, and training with bigger datasets did not enhance prediction accuracy. Reducing the number of hidden layer perceptrons from 100 (the default value) to 9 had no effect on the classifier’s performance. Even a 15 amino acid peptide segment composition gives a high degree of uniqueness and highly scalable intensity for survivin-peptide interaction. Survivin has a non-globular structure with no deep binding pockets. Survivin, despite this, may selectively associate with and avoid a wide range of peptides with distinct amino acid compositions that do not require a certain level of net charge or hydrophobicity.

### Oligomerization state of survivin

We investigated the oligomerization state of survivin as a function of concentration to see how it related to the different types of experiments. Survivin was in the expected dimeric form at 0.6 mg/mL (35.7 μM monomeric concentration), according to size exclusion chromatography-coupled small-angle X-ray scattering (SEC-SAXS) data (Figure S4). The particle molecular weight (MW) is estimated to be between 32.7 and 33.8 kDa, which is nearly double the theoretical MW of a survivin monomer (16.8 kDa). Based on Guinier analysis^41^ (Figure S4B), the radius of gyration is calculated to be 2.88 ± 0.02 nm (2.94 nm as defined by the p(r) function, Figure S4C), corresponding to a more elongated particle than monomeric survivin (Figure S4, Table S7). The Kratky plot^42^ demonstrates that the survivin structure is partially flexible (Figure S4D). At 0.8 mg/mL survivin concentration, batch SAXS experiments revealed a R_g_ of 2.91 nm, an MW range of 34.2–38.1 kDa. Batch experiments also indicated a concentration dependence of the size parameters (Figure S4F). The SEC-SAXS contrast curve was modeled using PDB entry 6SHO^43^ and yielded a close agreement with a χ^2^ value of 1.29 (Figure S4E). In addition, the *ab initio* generated model envelope superposes well with the 6SHO^43^ crystal structure.

Microscale thermophoresis experiments indicated a clear transition at 1.4 ± 0.4 μM when labeled survivin was titrated with unlabeled survivin, which we interpreted as a monomer-dimer transition (Supporting online text, Figure S5, Table S8). At high survivin concentration, a systematic contrary trend was observed similar to the DVD-Actin/VCA′∗ interaction,^44^ and the linear component of the Bayesian model successfully captured this trend.^45^ The positive interparticle effects in the SAXS data that were seen at high survivin concentrations may be what causes this contrary tendency. The survivin concentration in the microarray experiment was 1 μg/mL (53.5 nM monomer) suggesting that survivin was present predominantly in monomeric form.

### Characterization of the survivin-PRC2 peptide interaction

**Figure 5:**
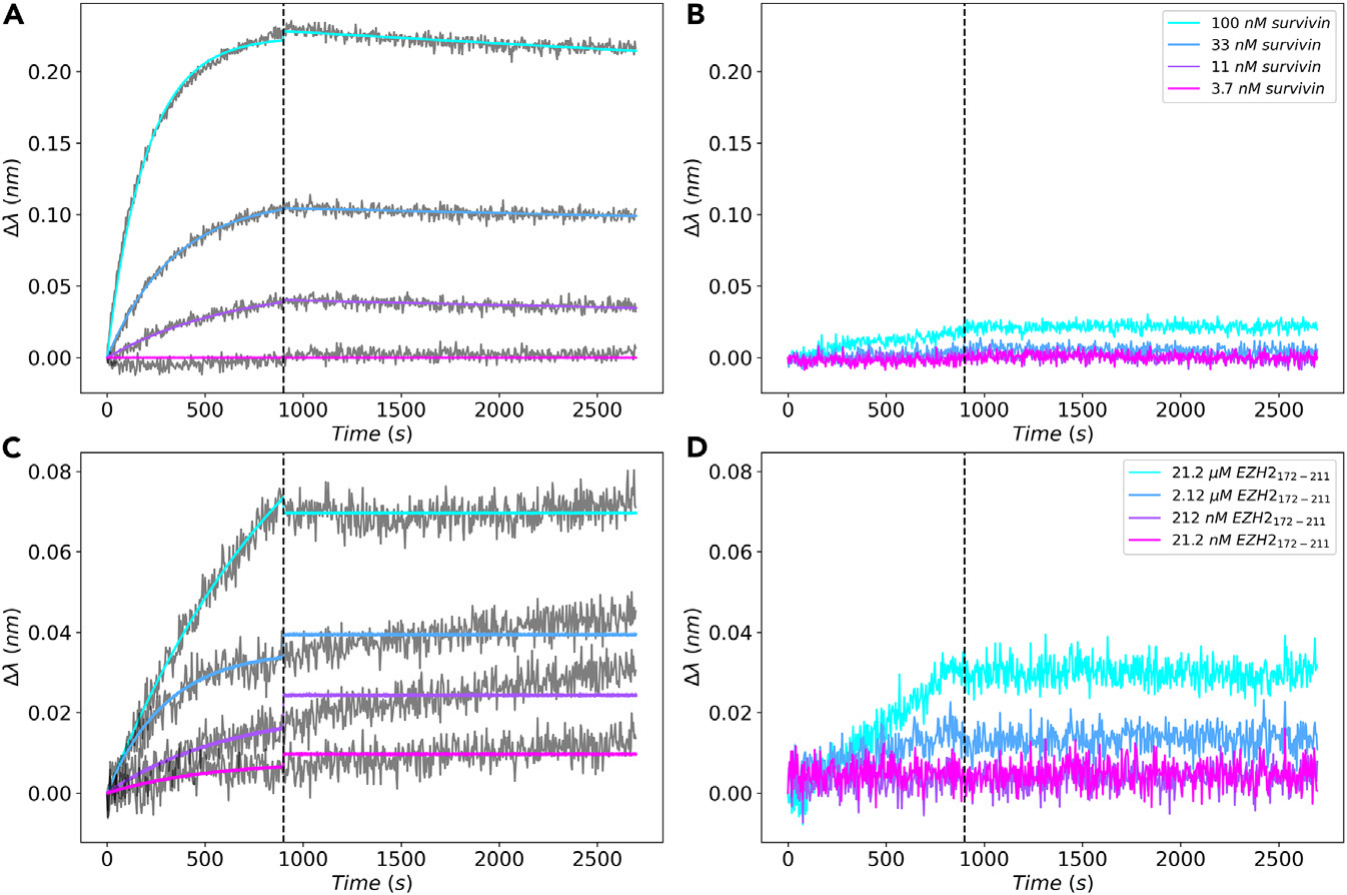
Biolayer interferometry analysis of the survivin-EZH2_172-211_ peptide interaction, related to Table S9 and Figure S6 (A) BLI experiment showing wavelength shift of biosensors covered by peptide EZH2_172-211_ upon survivin incubation in the 3.7–100 nM concentration range. The raw data are shown in *gray,* and the mean posterior predictions are shown in the *magenta-cyan* color scale corresponding to increasing survivin concentration. (B) Wavelength shift profile from control biosensors (not containing the peptide) for corresponding experiments in (A). The raw data are shown with the *magenta-cyan* color scale corresponding to increasing survivin concentration. (C) Wavelength shift profile of biosensors covered with survivin upon EZH2_172-211_ peptide incubation (in concentration range 21.2 nM–21.2 μM). The raw data are shown in *gray* and the mean posterior predictions are shown in the *magenta-cyan* color scale corresponding to increasing EZH2_172-211_ concentration. The wavelength shift profiles were monitored over association (0–900 s) and dissociation (900–2650 s) periods. (D) Wavelength shift profile from control biosensors (not containing survivin) for corresponding experiments in (C). The raw data are shown with the *magenta-cyan* color scale corresponding to increasing EZH2_172-211_ concentration.

To mimic the conditions of the microarray experiment and provide kinetic analysis of the interactions, we performed BLI experiments. In one of the experiments, the sensor surface was covered with the EZH2_172-211_ peptide and survivin served as analyte. The thickness of the biolayer is related to the BLI wavelength shift trace (Δλ(t)) profiles, which in turn depends on the peptide-survivin interaction. Survivin interacted with EZH2_172-211_-coated sensors at concentrations of 100, 33, and 11 nM. (Figure 5A). In contrast, sensors lacking the EZH2_172-211_ peptide exhibited an interference shift that was either stable over time or slightly increases (Figure 5B). In a second set of experiments, survivin was bonded to the biosensor during a reverse experiment, and EZH2_172-211_ peptide was the analyte (Figure 5C). Due to its smaller size than survivin, the peptide introduced a lower wavelength shift and was more likely to interact with the survivin-free biosensor at sub-micromolar concentrations (Figure 5D). A quantitative difference could still be observed when survivin was attached to the biosensor. In this light, it is not unexpected that a wide range of fluorescence intensity was visible in the microarray experiment despite the rather rigorous washing procedures, which did not remove survivin from the array surface. A Bayesian 1:1 model was developed to simulate the association and dissociation phases of the BLI experiments. The mean posterior predictions are shown in Figure 5A and 5C, and residual plots are shown in Figure S6. The distribution of posterior model parameters is described in Table S9. It indicates nanomolar interaction when the apparent dissociation constant is well defined.

We selected four peptides (Table 3) with high survivin-associated fluorescence intensity in the peptide microarray representing four subunits (EZH2_172-211_, EED_128-152_, SUZ12_573-597_, and JARID2_170-194_). To describe the interaction of the peptides in more detail and to identify eventual modulations of structure and dynamics of survivin, we used solution NMR spectroscopy in the next step. To this end, we measured uniformly labeled [*U*-^15^N]-survivin and obtained 2D [^15^N,^1^H]-NMR spectra of reasonable quality (Figure S7). As the quality of the 2D spectra and the 3D backbone resonance assignment spectra was not of sufficient quality for a complete *de novo* sequence-specific resonance assignment, we employed an approach using a previously reported sequence-specific backbone resonance assignment of a shorter survivin_1-117_ construct (BMRB: 6342) in conjunction with HNCA and HNCO experiments yielding a ∼84% complete assignment of the protein backbone resonances (Figure S7). When the obtained secondary chemical shifts of the Cα groups were compared to the previously deposited assignments, no notable changes in secondary structure elements were observed, particularly for the C-terminal residues in the longer full-length construct, where no stable helix formation could be detected (Figure S7C). The strong signal intensity for the residues in this area also reflects the highly dynamic character of this region, which is in contrast to the observation for the full-length X-ray structures revealing a stable helix.^43^^,^^46^^,^^47^

**Figure 6:**
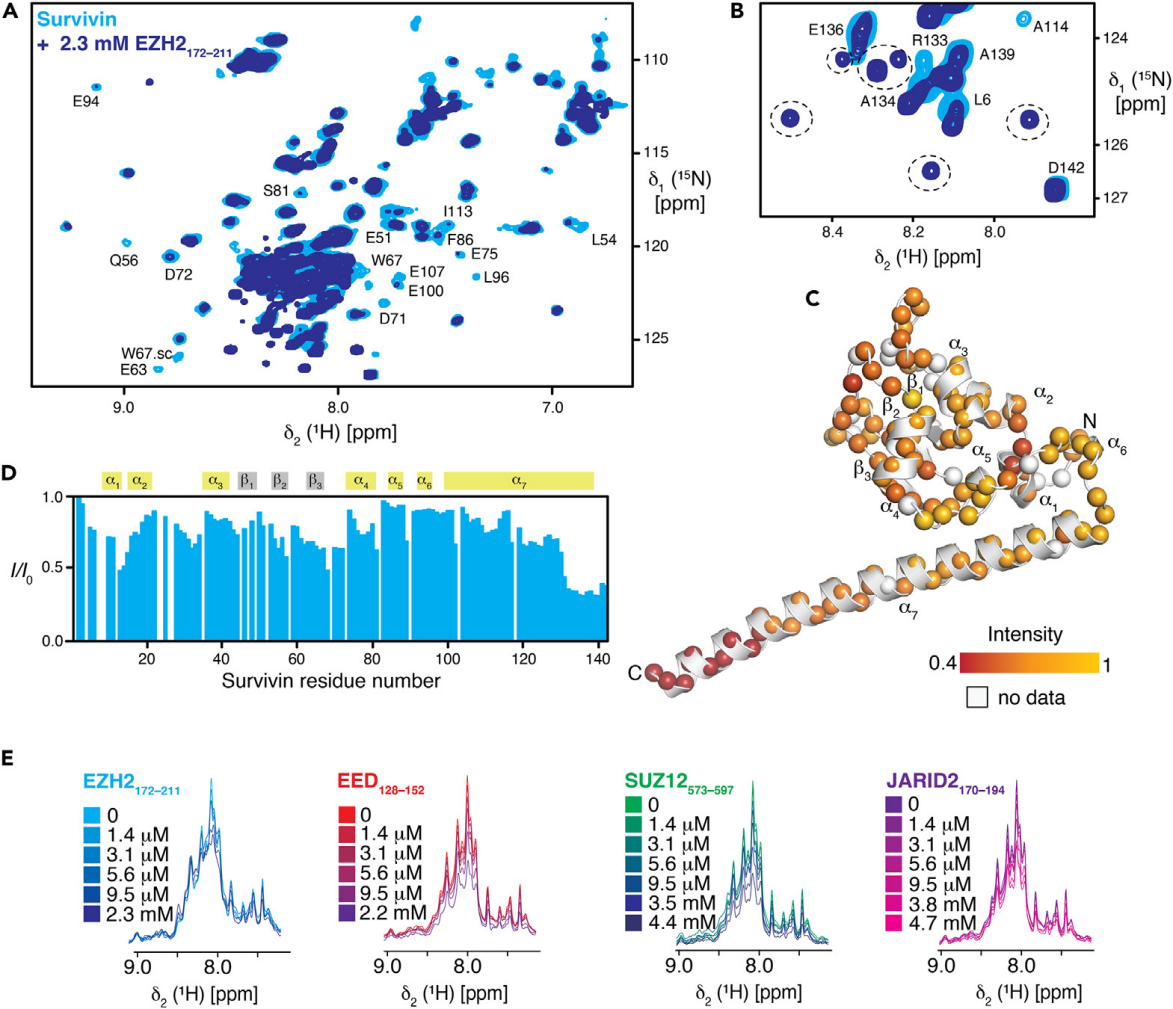
NMR titration of survivin with peptides derived from different PRC2 subunits, related to Tables S8 and S10 and Figures S4–S8 (A) Overlay of 2D [^15^N,^1^H] NMR spectra of 300 μM [*U*-^15^N]-survivin in the absence (*cyan*) and presence (*blue*) of 2.3 mM EZH2_172–211_ peptide measured at 298 K in NMR buffer. Characteristic peaks are annotated. “sc” refers to tryptophan indole resonances. (B) Zoom-in into the NMR-spectra overlay at higher contour lines. Characteristic peaks are annotated. Newly appearing resonances at high peptide concentration are encircled. (C) Intensity changes upon EZH2_172–211_ interaction were plotted on the survivin structure (PDB: 3UEF; only a single monomer is shown) by the indicated color gradient. The amide moieties are shown as spheres. (D) The ratio of the individual peak intensities in the presence of the EZH2_172–211_ peptide plotted against the surviving residue number. (E) δ_1_[^15^N]-1D cross-sections from 2D [^15^N,^1^H] NMR spectra of a titration of [*U*-^15^N]-survivin in NMR buffer with variable concentrations as indicated of the following peptides: EZH2_172–211_ (blue), EED_128–152_ (red), SUZ12_573–597_ (green), and JARID2_170–194_ (purple).

We then tested the interaction of [*U*-^15^N] survivin with increasing amounts of PRC2-derived peptides using these sequence-specific assignments. Titrating an increasing amount of EZH2_172-211_ peptide revealed mainly changes in signal intensity, indicating an interaction on either the slow or intermediate regime on the NMR timescale with kinetic exchange rate constants ranging from 1 to 1000 s^−1^ (Figure 6A). Aside from the outlined effects on the global signal intensity upon peptide binding, we observed the appearance of new resonances at high peptide concentrations for a subset of residues, indicating an interaction in the slow exchange regime compatible with a strong binding in the low nanomolar range or below (Figure 6B).

Analyzing the NMR signal attenuation in a residue-specific manner revealed that, while the most of the survivin resonances are affected, specific regions show the largest effects (Figures 6C and 6D): parts of the central β-sheet with strands β_2_ and β_3_, helices α_1_ and α_4_, and the most pronounced effect for the C-terminal part of helix α_7_. On one hand, the signal attenuation near the β-sheet could indicate the direct interaction site, which would not be surprising given that this region has been previously observed to be interacting with bound peptides.^47^^,^^48^^,^^49^ On the other hand, the large effect on the C-terminus may indicate more pronounced stabilization of the long helix observed in these structures, e.g., the complex with borealin and INCENP contains a stabilized coiled-coil structure with the C-terminus of survivin.^3^ At the current stage, we cannot rule out an alternate scenario for the strong effect on this part of the protein, such as a direct interaction with the peptide or a modulation of the survivin monomer-dimer equilibrium. Nevertheless, the observation of a new subset of survivin peaks upon high peptide concentrations, which possibly represents the helical state of these residues, is a plausible indication for C-terminal helix stabilization.

To qualitatively analyze the effects of a variety of different peptides, we compared 1D traces from 2D [^15^N,^1^H]-NMR spectra and all tested peptides show signal attenuations (Figure 6E). To quantify these observations, we analyzed the signal attenuation on a global scale (Figure S8). The total signal intensity decreased due to the interaction with the EZH2_172-211_ peptide and the onset of this transition appears to be the lowest of the compared peptides at critical peptide concentration of 2.1 μM. (Figure S6, Table S10) Notably, this peptide concentration is substantially lower than the concentration of survivin (300 μM as monomer), suggesting that this transition is unlikely to be mediated by interactions with a well-defined stoichiometry. Survivin alone at 6.4 mg/mL concentration (381 μM monomer) had a R_g_ of 3.2 nm with an estimated MW range 38.1–43.3 kDa in the batch SAXS experiments (Figure S4F) and the Guinier approximation started to break down due to attractive interparticle interactions. To facilitate this transition, the peptide may recruit survivin or act as a trigger or nucleus for a phase transition. Although the survivin concentration is much higher in the NMR than in the BLI experiment and thus cannot be used to quantify a binding in the sub-nanomolar range, the observation of a slow exchange regime agrees with the obtained BLI data. Overall, the NMR data together with the microarray and BLI experiments indicate direct interactions in line with the observed colocalization and possible functional links on the cellular level.

Initially, we expected that the interaction may be solely determined by specific pattern of relatively short-range bonds including hydrogen bonds, ion bridges, van der Waals forces, and hydrophobic shape complementarity, instead the large effects observed for diverse parts of survivin might point to either binding promiscuity and/or structural and dynamical adaptations of survivin. This interpretation is completely in line with the previous observation of a stabilized helix α_7_ by co-crystallization^43^^,^^46^^,^^47^ as well as the usage of more specific methyl groups to dock an interacting peptide in a previous NMR study.^50^ We showed a lack of specific surface interactions in the NMR experiments and a large sequence variety of peptides interacting with survivin in the peptide microarray experiment. As such, these interactions did not appear to primarily depend on the primary structure of peptides.

### Protein-protein docking with the SANT1 domain of EZH2 points to its inhibition of methyltransferase activity

**Figure 7:**
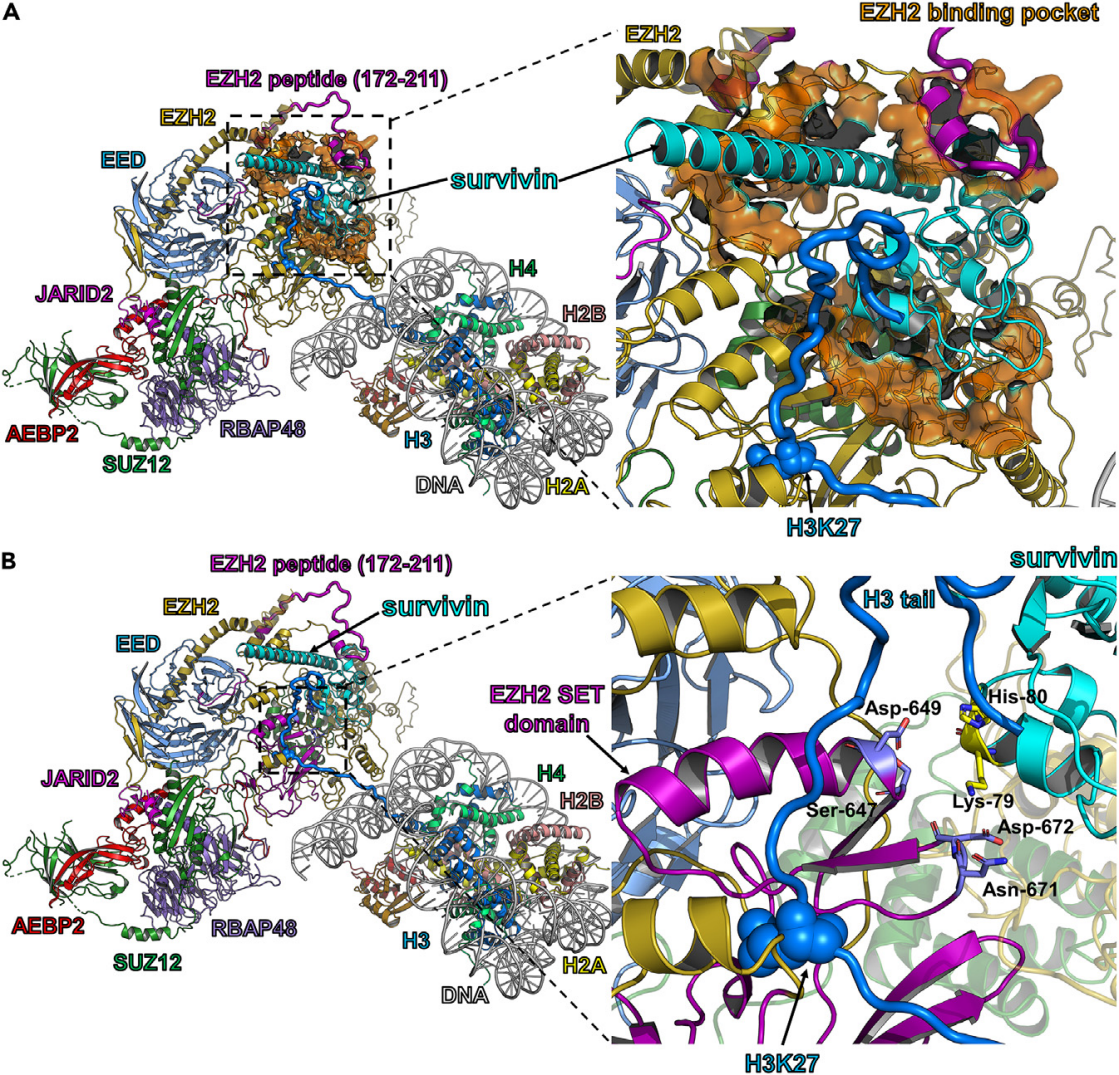
Survivin-dependent molecular mechanism of preventing PRC2 from methylating H3K27, related to Table S10 and Figures S9–S11 Histone H3 tails are shown in dark blue and survivin is shown in turquoise. (A) The EZH2 subunit forms a binding pocket to accommodate the survivin molecule. The EZH2 binding pocket is shown in the surface representation. (B) Survivin directly interacts with the catalytic SET domain of the EZH2 subunit and may disrupt the enzymatic activity required for trimethylation of H3K27.

To investigate the molecular mechanism of survivin interaction with the PRC2 complex, we performed protein-protein docking based on the experimental structures of human survivin^47^ and the PRC2 complex.^51^ The missing residues of the EZH2 subunit from the PRC2 complex were modeled as described in the STAR Methods, which resulted in a predicted complex structure of survivin bound to the EZH2 subunit (Figure 7A). Our model suggests that the SANT1 domain of EZH2 (residues 159 to 250) plays a critical role in forming the binding pocket to accommodate a long C-terminal α-helix of survivin. This is consistent with our peptide microarray experiments which show that the region (residues 172 to 211) from the SANT1 domain exhibits the strongest interaction with survivin (Figure 2). The short-range interactions between survivin and the binding pocket of the EZH2 SET domain are apparent from this docking model (Figure 7B). Specifically, the positively charged survivin residues Lys-79 and His-80 form salt bridges with the negatively charged EZH2 residues Asp-672 and Asp-649, respectively. These residues of the EZH2 SET domain are involved in the catalysis of H3K27 methylation,^52^ and will inevitably be affected by the interaction with survivin. Thus, we propose that this interaction between survivin and the catalytic SET domain of EZH2 disrupts its methyltransferase active site and abrogates trimethylation of H3K27 in a fashion described for small-molecular inhibitors of EZH2.^53^

### Conclusions

Survivin and PRC2 are functionally related and colocalize in human chromatin. In a peptide microarray assay, specific peptides derived from PRC2 subunits attracted survivin at low concentrations and formed a complex with non-detectable dissociation. These interacting peptides were frequently from critical functional areas of PRC2 subunits, which included catalytic, pocket-forming, and disease-associated amino acid residues. We demonstrated that predicting survivin interactions by associating peptide composition with colocalization of interacting peptide is a promising strategy, and the prediction reveals a complex, but robust pattern.

Even in the absence of a well-defined 3D structure, several peptide sections identified by atom composition can be combined into a selective protein-protein targeting system. Our docking analysis reinforces converging lines of experimental evidence: 1) ChIP analysis indicates that survivin and PRC2 subunits are frequently colocalized in the chromatin; 2) Survivin is known to interact with histone H3 tail; 3) PRC2 modifies histone H3 tails and the relative orientation and position of the subunits in the PRC2-nucleosome complex is not arbitrary; 4) The continuous region that interacts with survivin is in a specific region of EZH2; 5) Survivin inhibition increases H3K27 trimethylation, which consequently affects transcription of the HOX-B gene cluster. Points 1,4, and 5 presented in this study and points 2–4 can only be compatible with a survivin-PRC2 complex where a monomeric survivin forms a bridge over a druggable surface of EZH2 and occludes it from efficient substrate access. Even though the precise atomic details of the occlusion are not addressed here, it predicts an inhibition of PRC2 function, as evidenced by point 5.

We described a hitherto unrecognized function of survivin as a natural inhibitor of EZH2 methyltransferase activity in primary T cells. The effect of survivin could be translated into two distinct biological roles in autoimmune disorders or cancer. Inhibition of EZH2 in CD4^+^ T cells via genetic or biochemical means increases polarization toward the T helper (Th)1 and Th17 pathways,^13^^,^^14^^,^^54^ which is consistent with the survivin-dependent phenotype of IFNγ-producing CD4^+^ cells and follicular helper cells^55^ that stimulate inflammation and autoimmunity. EZH2 inhibition reduces viability in cancer cells^8^^,^^9^ and promotes signals that aid antitumor responses. EZH2 deficiency in the regulatory T cells increases tumor-infiltrating T cells frequency and strengthens tumor control, indicating that an inflammatory phenotype supports antitumor immunity.^17^^,^^56^ These findings add a new piece of knowledge to the molecular mechanisms bridging immune responses and cancer.

### Limitations of the study

The study lacks specificity regarding YM155’s inhibitory effect, and subsequent research has uncovered YM155’s side effects and secondary targets. This has been extensively discussed earlier.^57^ The study did not explore the potential association between apoptosis and PRC2 and survivin. Additionally, the study did not investigate alternative factors, such as the primary and secondary structure of peptides, for predicting peptide-survivin interaction. The study did not address the question why the atom composition of the peptides has good predictive value. The study relied solely on empirical models and did not provide direct evidence of the atomic-level reaction mechanism. The NMR and SAXS experiments were conducted at high concentrations, which may not accurately reflect the concentrations in cells. The validity of the K_D_ value determined in the BLI analysis may only be applicable if binding occurs through a 1:1 mechanism, whereas the NMR experiment revealed a substochiometric effect of the peptide. The interpretation of the MST experiment depends on the assumption that the labeled survivin molecules have the same characteristics as the unlabeled ones and that their tendency to form dimers remains unaffected. The docking model used in the study requires experimental validation, and other atomistic mechanisms may also account for survivin’s inhibitory effect.

## STAR★Methods

### Key resources table

**Table:** 

REAGENT or RESOURCE	SOURCE	IDENTIFIER
**Antibodies**		

Survivin - ChIPseq, CD4	Santa Cruz Biotechology	Polyclonal rabbit IgG 10811; RRID: AB_355684
H3-K27-me3 – ChIPseq, CD4	Diagenode	C15410195; RRID:AB_2753161
H3-K27-ac – ChIPseq, CD4	Diagenode	C15410196; RRID:AB_2637079
Anti-CD3	Sigma-Aldrich	OKT3; RRID: AB_2619696

**Bacterial and virus strains**		

*E. coli* BL21(DE3)star	Merck	69450

**Biological samples**		

Human CD4 + T cells, healthy controls	This paper	N/A

**Chemicals, peptides, and recombinant proteins**		

YM155, serpantronium bromide	Selleck Chemicals	S1130
Lymphoprep	Axis-Shield PoC As	LYS3773
RPMI	Gibco	21870-070
b-mercaptoethanol	Gibco	31350-010
Glutamax	Gibco	A12860-01
Gentamicin	Sanofi-Aventis	stock, 40 mg/ml
Fetal bovine serum	Sigma-Aldrich	F7524
Recombinant IFNγ	Peprotech	SKU: 300-02
Concanavalin A (ConA)	MP biomedicals	11492082
Lipopolysaccharide (LPS)	Sigma-Aldrich	L2880 *E. coli*, O111B4
EZH2_172-211	Biomatik	N/A
EED_128-152	Biomatik	N/A
SUZ12_573-597	Biomatik	N/A
JARID2_170-194	Biomatik	N/A
Fluorescence labeling kit	Labelling kit Green-MALEIMIDE (cysteine reactive)	MO-L005, NanoTemper technologies
MST capillaries	Monolith Capillaries	MO-K022, NanoTemper technologies

**Critical commercial assays**		

PEPperCHIP Peptide Microarray	PEPperPRINT Gmbh	www.pepperprint.com
Positive selection of human CD4	Invitrogen	11331D
Chromatin isolation	Qiagen	EpiTect ChIP OneDay
mRNA extraction	Norgen	Total micro mRNA kit
Lysis buffer for immunoprecipitation	Pierce	87787
Protease inhibitors	Roche	Complete Mini
Dynabeads protein G IP beads	Invitrogen	10007D

**Deposited data**		

Survivin ChIP-seq, human CD4^+^ T cells	https://doi.org/10.1016/j.isci.2022.105526	GEO: GSE190354
Survivin ChIP-seq, human CD4^+^ T cells, YM155-treated	https://doi.org/10.1016/j.isci.2022.105526	GEO: GSE198292
H3K27me3 ChIP-seq, human CD4^+^ T cells, YM155-treated	This paper	GEO: GSE216818
H3K27ac ChIP-seq, human CD4^+^ T cells, YM155-treated	This paper	GEO: GSE216817
RNAseq, human CD4^+^ T cells	https://doi.org/10.1016/j.isci.2022.105526	GEO: GSE190349
RNAseq, human CD4^+^ T cells YM155-treated, 72h	https://doi.org/10.1016/j.isci.2022.105526	GEO: GSE190351
Survivin peptide microarray	This paper	https://doi.org/10.5281/zenodo.7774524
Survivin SAXS data	Deposit at SASBDB	SASDR86
Survivin structures	From PDB	6sho/3uef
CryoEM PRC2 structure	From PDB	6wkr
Human Survivin	BMRB	6342

**Recombinant DNA**		

pHis8-survivin plasmid	Obtained from Verdecia lab	https://doi.org/10.1038/76838

**Software and algorithms**		

DESeq2 (v.1.4.0)	Bioconductor	https://bioconductor.org/packages/release/bioc/html/DESeq2.html
STARaligner	Dobin, et al. 2013,^58^ Github	https://github.com/alexdobin/STAR
HOMER	Heinz et al.^59^	http://homer.ucsd.edu/homer/
ENSEMBL regulatory build	v103, 2020	http://www.ensembl.org/info/docs/funcgen/regulatory_build.html
ENCODE	v5, 2020	https://screen.encodeproject.org/ https://api.wenglab.org/screen_v13/fdownloads/GRCh38-ccREs.CTCF-only.bed file
GeneHancer	v4.4	https://www.genecards.org/GeneHancer_version_4-4
Galaxy Suite		https://usegalaxy.org/
Cistrome Galaxy	CEAS v0.9.8; accessed November 1, 2020	http://cistrome.org/ap/root
Bedtools Suite	Github, accessed accessed 01feb2021–15apr 2021	https://github.com/arq5x/bedtools2
ReMap database	accessed November 15, 2020	http://remap.univ-amu.fr/
ReMap enrich	Github accessed November 15, 2020	https://github.com/remap-cisreg/ReMapEnrich
Gene Ontology Biological Processes	GSEA	http://www.gsea-msigdb.org/gsea/msigdb/human
ATSAS package for SAXS analysis	EMBL	Version 3.0.0
ChimeraX	UCSF	Version 1.2.5
Monolith NT.115 instrument /software	NanoTemper Technologies	N/A
MODELLER	Benjamin Webb, Andrej Sali	Version 10.3
HDOCK	Yan et al.^60^	http://hdock.phys.hust.edu.cn/
TopSpin4.0.4	Bruker BioSpin	4.0.4
NMRPipe	Delaglio et al.^61^	https://www.ibbr.umd.edu/nmrpipe/install.html
mddNMR2.6	Jaravine et al.^62^	https://mddnmr.spektrino.com/
Cara	Keller^63^	https://wiki.cara.nmr.ch/FrontPage
PyMol	Schrödinger^64^	https://pymol.org/2/
POTENCI	Nielsen and Mulder^65^	https://st-protein02.chem.au.dk/potenci/
ThermoBayes	Anindya et al.^45^	https://github.com/Katona-lab/ThermoBayes
BayesLayerAnalyst	This paper	https://github.com/Katona-lab/BayesLayerAnalyst
"Bayesian NMR Titration Analyzer" or "BNTRA"	This paper	https://github.com/Katona-lab/BNTRA
pymc3	Salvatier et al.^66^	https://www.pymc.io/projects/docs/en/v3/index.html
Peptide microarray analysis	This paper	https://doi.org/10.5281/zenodo.7774524

### Resource availability

#### Lead contact

Further information and requests for resources and reagents should be directed to and will be fulfilled by the lead contact, Gergely Katona (gergely.katona@gu.se)

#### Materials availability

This study did not generate new unique reagents.

### Experimental model and subject details

Blood samples of 24 rheumatoid arthritis (RA) patients were collected at the Rheumatology Clinic, Sahlgrenska Hospital, Gothenburg. All RA patients fulfilled the EULAR/ACR classification criteria^67^ and gave their written informed consent prior to the blood sampling. The study was approved by the Swedish Ethical Evaluation Board (659-2011) and was performed in accordance with the Declaration of Helsinki. The trial is registered at ClinicalTrials.gov with ID NCT03449589.

CD4^+^ cells from healthy controls were used for ChIP-seq, RNA-seq after YM155 treatment.

### Method details

#### Isolation and stimulation of CD4^+^ cells

Human peripheral blood mononuclear cells were isolated from venous peripheral blood by density gradient separation on Lymphoprep (Axis-Shield PoC As, Dundee, Scotland). CD4^+^ cells were isolated by positive selection (Invitrogen, 11331D), and cultured (1.25×10^6^ cells/ml) in wells coated with anti-CD3 antibody (0.5 mg/ml; OKT3, Sigma-Aldrich, Saint Luis, Missouri, USA), in RPMI medium supplemented with 50 μM β-mercaptoethanol (Gibco, Waltham, Massachusetts, USA), Glutamax 2 mM (Gibco), Gentamicin 50 μg/ml (Sanofi-Aventis, Paris, France) and 10% fetal bovine serum (Sigma-Aldrich) at 37°C in a humidified 5% CO_2_ atmosphere. Cells were treated with survivin inhibitors serpantronium bromide, YM155^22^ (YM155, Selleck Chemicals, Houston, TX), as indicated. The cells were stimulated with recombinant IFNg (50 ng/ml; Peprotech, Cranbury, NJ, USA) during the last 2 h and harversted for RNA-seq. Supernatants were used to measure cytokine levels.

#### RNAseq experiments

RNA from the CD4^+^ cell cultures was prepared using the Norgen Total micro mRNA kit (Norgen, Ontario, Canada). Quality control was done by Bioanalyzer RNA6000 Pico on Agilent2100 (Agilent, Santa Clara, CA, USA). Deep sequencing was done by RNAseq (Hiseq2000, Illumina) at the Science for Life Laboratory, Huddinge, Sweden. Raw sequence data were obtained in Bcl-files and converted into fastq text format using the bcl2fastq program from Illumina.

#### Chromatin immunoprecipitation (ChIPseq)

For ChIP-seq analysis, CD4^+^ cells isolated from 12 women were stimulated with concanavalin A (ConA, 0.625 μg/ml, Sigma-Aldrich), and lipopolysaccharide (LPS) (5 μg/ml, Sigma-Aldrich) for 72 h and pooled in 4 independent samples for DNA purification. After shearing and pre-clearing of chromatin, 1% of the sample was saved as an input. The pre-cleared chromatin was incubated with 2 μg anti-Survivin (10811, Santa Cruz Biotechnology). For ChIPseq targeting histone modifications CD4^+^ cells from 3 healthy women were stimulated with concanavalin A and LPS and treated with sepantronium bromide, YM155, 0 or 10 ng/ml, for 24 h. The chromatin was pre-cleared on the beads coated with Sepharose A and incubated with anti-H3K27ac (C15410196, Diagenode), or anti-H3K27me3 (C15410195, Diagenode). The cells were cross-linked and lysed with the EpiTect ChIP OneDay kit (Qiagen), as recommended by the manufacturer. After sonication to shear the chromatin, cellular debris was removed by pelleting. After pre-clearing, 1% of the sample was saved as an input fraction and used as background for nonspecific chromatin binding. Pre-cleared chromatin was incubated with 2 μg of anti-survivin (10811, Santa Cruz Biotechnology, Santa Cruz, CA, USA). The immune complexes were washed, the cross-links were reversed, and the DNA was purified with the EpiTect ChIP OneDay kit (Qiagen) as recommended by the manufacturer. The quality of purified DNA was assessed with TapeStation (Agilent, Santa Clara, CA, USA). DNA libraries were prepared with ThruPLEX (Rubicon) and sequenced with a Hiseq2000 sequencing system (Illumina) according to the manufacturer’s protocols. Bcl-files were converted and demultiplexed to fastq with bcl2fastq (Illumina).

#### Production and purification of survivin

Survivin in pHIS8^46^ was expressed in *E. coli* BL21(DE3) star cells (Merck). Luria Bertani (LB) medium was used, and expression was induced with 0.5 mM IPTG and incubated for 4 h at 30°C.^68^ Cells were harvested at 6,000 × g for 30 minutes and resuspended in lysis buffer containing 50 mM Tris pH 7.5, 300 mM NaCl, 20 mM imidazole, 0.25 mM Pefabloc (Sigma-Aldrich), 20 mg/ml DNAse (Sigma-Aldrich) and 0.2 mg/ml lysozyme (Sigma-Aldrich). The cells were lysed by sonication with an amplitude of 30 A for 10 minutes (10s on/30s off) by a Q700 sonicator (Qsonica). Survivin was purified by immobilized metal ion chromatography (IMAC) with a 5 ml HisTrap FF (GE Healthcare) column followed by overnight dialysis in 50 mM Tris pH 7.5, 150 mM NaCl and 1 mM DTT (survivin SEC buffer) and gel filtration using a Superdex 75 100/300GL column (GE Healthcare).

#### Peptide microarray experiments

A peptide microarray was designed using the protein sequences from: Cdk1 (P06493), KAT2A/GCN5 (Q92830), SPI1/PU1 (P17947), SUZ12 (Q15022), EED (O75530), JADE3 (Q92613), DIABLO/SMAC (Q9NR28), BOREALIN (Q53HL2), INCENP (Q9NQS7), SGOL1 (Q5FBB7), SGOL2 (Q562F6), EZH2 (Q15910), JARID2 (Q92833), Histone H3 (P68431), AURORAKB (Q96GD4), JADE1 (Q6IE81), JTB (O76095), EVI5 (O60447), RAN (P62826), USP9X (Q93008), C-IAP1 (Q13490), STAT3 (P40763), BRUCE/APOLLON (Q9NR09), XPO1 (O14980), CDX2 (Q99626), Msx2 (P35548), RBM15 (Q96T37), PHF21A (Q96BD5), PHF8 (Q9UPP1), DIDO (Q9BTC0), JADE2 (Q9NQC1) and HASPIN (Q8TF76). The Uniprot ID is shown in parenthesis. The protein sequences were divided into 15 amino acid long peptides with a 10 amino acid overlap. To investigate background interactions with the protein-derived peptides that could interfere with the main assays, one of the PEPperCHIP Peptide Microarrays (PEPperPRINT Gmbh) was pre-staining with the secondary 6x His Tag Antibody DyLight680 antibody at a dilution of 1:1000 and with the monoclonal anti-HA (12CA5)-DyLight800 control antibody at a dilution of 1:1000. Subsequent incubation of other peptide microarray copies with survivin at a concentration of 1 μg/ml in incubation buffer was followed by staining with the secondary 6x His Tag Antibody DyLight680 antibody (Rockland Immunochemicals, Pottstown, PA, USA) and the monoclonal anti-HA (12CA5)-DyLight800 control antibody (Rockland Immunochemicals, Pottstown, PA, USA). The read-out was performed by scanning intensities of 7/7 (red/green). As an internal quality control, HA and His tag control peptides were stained simultaneously to confirm assay quality and to facilitate grid alignment for data quantification. The LI-COR Odyssey Imaging System was used for read-out, and the PepSlide Analyzer was used for quantification of spot intensities and peptide annotation. Spot intensity quantification and peptide annotation were performed using 16-bit gray scale tiff files at scanning intensities of 7/7, which have a greater dynamic range than the 24-bit colorized tiff files shown in Figure S1.

#### SAXS experiments

The His-tag of survivin was removed by thrombin digestion and a subsequent gel filtration purification step. The SAXS experiments were carried out at the BM29 beamline of the European Synchrotron Radiation Facility. A Pilatus 1M detector in vacuum was used to record the q range of 0.03–5 nm^−1^ with 1 s exposure per frame. A Superdex 75 100/300GL column was used for SEC-SAXS, with a 500 μl injected volume of 3 mg/ml survivin solution (50 mM Tris pH8, 150 mM NaCl, 1 mM DTT). The flow rate was 0.6 ml/min and the sample temperature was 10°C. The batch mode experiments were performed with the automated sample changer of the beamline and filtered samples.

#### MicroScale thermophoresis (MST) experiments

Survivin was chemically labelled with the MO-L005 Monolith™ Protein Labelling Kit GREEN-MALEIMIDE (Cysteine Reactive) from NanoTemper Technologies (Germany) and was used at 100 nM concentration. The MST experiments were performed in 50 mM Tris pH 8.0, 150 mM NaCl, 1 mM DTT and 0.05% Tween. The unlabelled survivin was diluted and titrated in the same buffer.

The green channel of a Monolith NT.115 (green/blue) instrument from NanoTemper Technologies (Germany) was used for MicroScale Thermophoresis experiments in accordance with their protocol. The following buffer was used for the serial dilution of unlabeled survivin: 50 mM Tris pH 8.0, 150 mM NaCl, 1 mM DTT, and 0.05% Tween. The experiments were carried out at room temperature (24°C). The labelled protein concentration was kept constant at 97 nM and the unlabelled survivin was used at concentrations from 23.2 nM to 761 μM.

The experiments were carried out using 20% MST power after serial dilution and incubation for 5 minutes. The MST traces were captured using the standard parameters: 5 s of MST power off. 30 s MST power on (for progress curve analysis) and 5 s MST power off.

#### Biolayer interferometry (BLI) experiments

EZH2_178-211_ peptide from previous peptide microarray experiments were used as immobilized ligands using amine reactive second-generation biosensor (AR2G, Fortebio, Fremont, CA, USA). Ethanolamine was used to quench unbound AR2G surface to prevent non-specific interactions. The biosensor was incubated in MQ water for 15 minutes before use in the Octet instrument (Fortebio, Fremont, CA, USA) with the following settings: equilibration in water for 60 s and 1000 rpm shaking, activation in EDC/s-NHS for 300 s at 1000 rpm, peptide immobilization in immobilization buffer pH 6.0 (Fortebio) for 900 s and 1000 rpm (50 μM for the peptide), quenching with 1 M ethanolamine for 300 s and 1000 rpm, baseline measurement in survivin SEC buffer for 600 s and 1000 rpm, association with survivin (100 nM, 33.3 nM, 11.1 nM, and 3.7 nM concentrations) as the experiment analyte for 600 s and 1000 rpm, and dissociation step in survivin SEC buffer for 1200 s and 1000 rpm. The assay was performed at 30°C. The reference wells and reference biosensors were subtracted.

For the reverse experiments, survivin (2.5 μM) was immobilized instead of the peptide and was exposed to EZH2_178-211_ peptide with 21.2 μM, 2.12 μM, 212 nM, 21.2 nM concentrations during the association phase for 600 s and 1000 rpm. Other phases in the reverse experiment follow the same parameters as in non-reverse experiments mentioned above.

#### Isotopic labeling and NMR titration experiments

Uniformly [*U*] labeled [*U*-^15^N] survivin was produced in H_2_O-based M9 medium^69^ containing ^15^NH_4_Cl as the sole nitrogen and in case of or [*U*-^15^N,^13^C] survivin *D*-(^13^C)-glucose was additionally added as the sole carbon source, respectively (Sigma-Aldrich). Expression was induced with 0.5 mM IPTG and continued for 20–22 h at 15°C. Cell harvesting and subsequent purification followed the same protocol as for unlabeled survivin.

NMR measurements were performed on Bruker (Fällanden, Switzerland) AvanceIII 800 MHz spectrometers running Topspin 3.5 or 3.6, respectively, equipped with cryogenically cooled triple resonance probes. All NMR experiments were performed in NMR buffer (50 mM Tris, 150 mM NaCl, 1 mM DTT, pH 7.5) and at 298 K. The sequence-specific backbone assignment of survivin_1-117_ was taken from previously published assignment,^50^ and transferred to the survivin_1-142_ construct used in the present study. Existing resonance assignments were confirmed, and additional resonances were assigned in a sequence-specific manner by the following experiments: [^1^H,^15^N]-TROSY HSQC,^70^ BEST-TROSY-type HNCA and HNCO experiments.^71^

The peptides were synthetized and purified to 95% by Biomatik. The peptides were titrated into ^15^N labelled survivin (0.3 mM). Standard 2D [^15^N,^1^H]-SOFAST-HMQC^72^ experiments were recorded with inter-scan delays of 200 ms and 1025 x 256 complex points for the ^1^H and ^15^N dimensions, respectively, for the titrations of the different peptides with [*U*-^15^N] survivin.

#### Modeling of survivin-PRC2 complex

The structure of survivin bound to the PRC2 complex has yet to be determined experimentally. We used the high-resolution cryo-electron microscopy (Cryo-EM) structure of PRC2 bound to a nucleosome (PDB ID: 6WKR)^51^ as a basis for our modeling. The structure of EZH2 is not complete. It lacks N-terminal residues 1–16, SANT1 domain residues 182–219, and residues 340–425 of the EZH2 protein (Figure S9A). Thus, we first built the missing regions of the EZH2 subunit via structural modeling using MODELLER v10.3^73^ and obtained a model with the full protein sequence of the EZH2 (746 residues) (Figure S9B). Figure S9C shows the superimposition of the final EZH2 model with the EZH2 structure for comparison. The X-ray structure of human survivin bound to the H3 tail (residues 1–5) was retrieved from the protein data bank (PDB ID: 3UEF).^47^

The binding mode between survivin and the PRC2 complex (bound with H3 tail residues 19–43) was predicted using the hybrid protein-protein docking software HDOCK.^60^ The default parameters were applied during the docking calculation. The resulting binding modes and docking conformations were ranked by the binding energy scores, and the top ten docking conformations were selected for further modeling purposes (Table S11). Then, we tried to build the missing regions of the H3 tail (residues 6–18) using MODELLER v10.3 (Figure S10). MODELLER could not build the model of H3 tail for nine out of ten docking modes and only the second docking conformation satisfied the set of conformational orientations and distance restraints. Therefore, the final binding mode between survivin and the PRC2 complex with a full H3 tail was generated based on the docking conformation 2 (Figure S11). The interaction residues between survivin and the PRC2 complex were defined if two non-hydrogen atoms were within a distance of less than 5.0 Å. Molecular structures were visualized using PyMol (Schrödinger).^64^

### Quantification and statistical analysis

#### RNAseq analysis

Mapping of transcripts was done using Genome UCSC annotation set for hg38 human genome assembly. Analysis was performed using the core Bioconductor packages in R-studio v.3.6.3. Differentially expressed genes in CD4^+^ cells treated with YM155 were identified using DESeq2 (v.1.26.0) with Benjamini-Hochberg adjustment for multiple testing.

#### ChIPseq analysis

The fastq sequencing files were mapped to the human reference genome (hg38) using the STAR aligner^58^ with default parameters apart from setting the alignIntronMax flag to 1 for end-to-end mapping. Quality control of the sequenced material was performed by FastQC tool using MultiQC v.0.9dev0 (Babraham Institute, Cambridge, U.K.). Peak calling was performed using the default parameters. Peaks were identified for enrichment of the immunoprecipitation fraction against input (adjusted p < 10^−5^) and annotated by HOMER software^59^ in standard mode to closest transcription start site (TSS). Peaks with overlapping localization by at least 1 nucleotide in several samples were merged and further on referred to as one peak.

#### ChIPseq metanalysis

To identify the transcription regulators in vicinity of survivin-ChIP peaks, we performed colocalization analysis of aggregated human ChIPseq datasets of 1034 transcriptional regulators using ReMap database (http://remap.univ-amu.fr/, accessed 15nov2020). Colocalization enrichment analysis was performed using ReMapEnrich R-script (https://github.com/remap-cisreg/ReMapEnrich, accessed 15nov2020). All comparisons were carried out using hg38 human genome assembly. Two-tailed p values were estimated and normalized by Benjamini-Yekutielli, maximal allowed value of shuffled genomic regions for each dataset (n = 15), kept on the same chromosome (shuffling genomic regions parameter byChrom = TRUE). Default fraction of minimal overlap for input and catalogue intervals was set to 10%. Bed interval files of survivin-ChIP peaks with 0, 1 and 10kb flanks were prepared. The dataset with 0kb flanks was compared with the Universe. Statistically significant enrichment of peak overlaps (q-value < 0.05, number >100) were selected. To compare the peaks identified in different H3 ChIP-seq datasets and after YM155 treatment, the tag normalization was used to obtain the total peak score and the tag counts for each ChIP-seq data set. To integrate ChIP-seq and RNA-seq data, the genomic regulatory elements located within the peaks were identified and a complete list of the connected genes was prepared using the GeneHancer database v5.9 (2021).

#### Classification of peptide microarrays by machine learning

The classification machine learning method was implemented using the scikit-learn python library.^74^ The number of atoms belonging to specific atom type categories was the feature describing each peptide. Table S6 was used to assign the atom types to the amino acids for this study. These were accumulated after translating each amino acid in the peptide to atom types. Ward’s method^75^ and Euclidean distances were used to perform hierarchical clustering. The 5388 peptides were divided into equally large training and test sets. The training set was classified as interacting (fluorescence intensity greater than zero) or non-interacting (fluorescence intensity equal to zero). Before the training, the features were standardized. The multi-layer perceptron classifier was trained using the default parameters of scikit-learn.

#### SAXS data analysis

The ATSAS package was used for data analysis.^76^ The ab initio model was produced by DAMMIF,^77^ refined with DAMMIN^78^ and averaged with DAMAVER.^79^ The data was deposited to SASBDB^80^ and the ID number is SASDR86.

#### Probabilistic modelling of MST progress curves

The MST data were analysed as previously described^45^ with the following modifications. The experiments modelled here had a preheating fluorescence recording of 5 s and total post-heating time trace of 25 s. A Bayesian model implemented in Python using the pymc3 library^66^ was described previously.^45^ By using an updated model, we assume that the interaction between two labelled survivin molecules is the same as the interaction between labelled and unlabelled survivin, and that labelled survivin is monomeric at a concentration of 97 nM. In this section, we summarize the reported model parameters and discuss the differences.

The reaction is assumed to occur between the monomers (*M*) and the dimer (*D*). Labelled and unlabelled survivin were assumed kinetically indistinguishable:

M+M=D

$$K_{d}=\frac{{\left[M\right]}^{2}}{\left[D\right]}$$

$$c_{n}=c_{n,unlabelled}+c_{labelled}$$

$$c_{n}={\left[M\right]}_{n}+2{\left[D\right]}_{n},$$

where *c*_*n*_ represents the total concentration of survivin molecules in the different capillaries. [*M*]_*n*_ and [*D*]_*n*_ represent the equilibrium concentrations. The degree of association (α) is obtained by solving the resulting quadratic equation and it is a function of *c*_*n*_ and *K*_*d*_:

$$\alpha_{n}=\frac{4c_{n}+K_{d}-\sqrt{{K_{d}}^{2}+8c_{n}K_{d}}}{4c_{n}}$$

*K*_*d*_ is a global variable with the same *a priori* expectations as previously described.^81^ *U* and *B* represent the total amplitude of the exponential processes for pure monomeric and pure dimeric survivin, respectively.

$$p\left(K_{d}|lower=1,upper=10^{6}\right)=\frac{1}{upper-lower}$$

$$p\left(U|\alpha =1,\beta =1\right)=\frac{\Gamma \left(\alpha +\beta \right)}{\Gamma \left(\alpha \right)\Gamma \left(\beta \right)}U^{\alpha -1}{\left(1-U\right)}^{\beta -1}$$

$$p\left(B|\alpha =1,\beta =1\right)=\frac{\Gamma \left(\alpha +\beta \right)}{\Gamma \left(\alpha \right)\Gamma \left(\beta \right)}B^{\alpha -1}{\left(1-B\right)}^{\beta -1}$$

The *a priori* assumptions of *U* and *B* are identical and are described by a flat β distribution. We ensure that fluorescence cannot increase beyond the initial level due to exponential (T-jump and thermophoretic) processes by assuming positive amplitudes (we leave that possibility open through a linear process though). At total survivin concentration *c*_*n*_, not completely dimerized survivin has intermediate exponential amplitudes *A*_*total.n*_ determined by the law of mass action, which are an entirely deterministic combination of the stochastic components listed above:

$$A_{total,n}=U+\left(B-U\right)\cdot \frac{4c_{n}+K_{d}-\sqrt{{K_{d}}^{2}+8c_{n}K_{d}}}{4c_{n}}$$

In our model, the experimental data is modelled as a part of a normal distribution, and the scale parameter *ε* is a single global variable with a lognormal *a priori* distribution. This choice is motivated by the belief that errors do not vary between capillaries.

$$p\left(\varepsilon |\mu =0,\tau =1\right)=\frac{1}{\varepsilon }\sqrt{\frac{\tau }{2\pi }}e^{-\frac{\tau }{2}{\left(\ln\;\varepsilon -\mu \right)}^{2}}$$

Each of the n thermophoretic progress curves is linked to a local random variable, and their models have one linear and two exponential components. Unlike in our previous study, the linear and exponential processes begin with IR laser irradiation:

$$L_{n}\left(t\right)=\nu_{0,n}\left(t\right)$$

$$E_{1,n}\left(t\right)=A_{1,n}e^{-\nu_{1,n}t}$$

$$E_{2,n}\left(t\right)=A_{2,n}e^{-\nu_{2,n}t}$$

Because *A*_*total,n*_ *= A*_*1,n*_*+A*_*2,n*_, the values can be linked together with a single, curve-associated random ratio parameter (*R*_*n*_) varying ranging from 0 to 1. *R*_*n*_ can be conveniently represented by a β distribution, and a slightly asymmetric prior expectation ensures that exponential processes with larger and smaller amplitudes are grouped together for comparison.

$$E_{1,n}\left(t\right)=R_{n}A_{total,n}e^{-\nu_{1,n}t}$$

$$E_{2,n}\left(t\right)=\left(1-R_{n}\right)A_{total,n}e^{-\nu_{2,n}t}$$

$$p\left(R_{n}|\alpha =2,\beta =1\right)=\frac{\Gamma \left(\alpha +\beta \right)}{\Gamma \left(\alpha \right)\Gamma \left(\beta \right)}R_{n}^{\alpha -1}{\left(1-R_{n}\right)}^{\beta -1}$$

Since the progress curves are normalized to 1 at t = −5.0 s, the final fluorescence level that the two exponential components approaches in an ideal experiment (i.e. an experiment without the linear component) is *I*_*n*_ *= 1 – A*_*total,n*_.

The remaining rate parameters were modelled as random variables using the uniform and lognormal likelihood functions with the following *a priori* parameters:

$$p\left(\upsilon_{0,n}|lower=-1,upper=1\right)=\frac{1}{upper-lower}$$

$$p\left(\upsilon_{1,n}|\mu =0,\tau =1\right)=\frac{1}{\nu_{1,n}}\sqrt{\frac{\tau }{2\pi }}e^{-\frac{\tau }{2}{\left(\ln\;\nu_{1,n}-\mu \right)}^{2}}$$

$$p\left(\upsilon_{2,n}|\mu =0,\tau =1\right)=\frac{1}{\nu_{2,n}}\sqrt{\frac{\tau }{2\pi }}e^{-\frac{\tau }{2}{\left(\ln\;\nu_{2,n}-\mu \right)}^{2}}$$

Before t = 0 s progress curves are modelled as constant at 1:

$$p\left(P_{n}\left(t\right)|\mu =1,\varepsilon \right)=\sqrt{\frac{1}{2\pi \varepsilon }}e^{-\frac{1}{2\varepsilon }{\left(P_{n}\left(t\right)-\mu \right)}^{2}}\text{,}$$

where μ and ε corresponds to the location and scale parameter of the normal distribution (one global scale parameter).

When *t* ≥ 0 s the progress curves are modelled as:

$$p\left(P_{n}\left(t\right)|\mu =L_{n}\left(t\right)+E_{1,n}\left(t\right)+E_{2,n}\left(t\right)+I_{n},\varepsilon \right)=\sqrt{\frac{1}{2\pi \varepsilon }}e^{-\frac{1}{2\varepsilon }{\left(P_{n}\left(t\right)-\mu \right)}^{2}}$$

The model was scaled by the Automatic Differentiation Variational Inference (ADVI)^82^ algorithm and before sampling the posterior parameter space, and 16000 samples were collected in four parallel chains using the No-U-Turn sampling algorithm (NUTS).^83^ The 3000 samples from the four chains were combined to yield 12000 samples for the final analysis.

#### NMR data analysis

NMR data were processed with a combination of TopSpin 4.1.4 (Bruker Biospin, Fällanden, Switzerland), NMRPipe^61^ and mddNMR2.6^62^ as well as analyzed with CARA.^63^ Secondary chemical shifts were calculated relative to the random coil values using the prediction software POTENCI.^65^ Further a weighting function with weights *1–2–1* for residues *(i-1)–i–(i+1)* was applied to the raw data.^84^

For the global analysis, the NMR signal strength was quantified by summing the 200 highest values in each 2D NMR spectrum to ensure that all peaks corresponding to the different amino acid residues in survivin are potentially covered. The titration curve was modelled using a Bayesian machine learning approach using the python library pymc3.^66^ The logistic function was chosen due to computational convenience. The most important parameter of this function was coined “critical peptide concentration (cpc)” and its logarithm was modelled using a uniform *a priori* distribution between −10 and 1.

$$p\left(\log_{10}\;cpc|lower=-10,upper=1\right)=\frac{1}{upper-lower}$$

The modelling errors related to the choice of function affect the analysis of different peptides similarly. Therefore, the relative magnitude of cpc in comparison is more reliable than the absolute magnitude of cpc itself. The concentration values were converted to dimensionless quantities by dividing with 1 mM and the NMR signal intensity was normalized for the concentration range studied.

A deterministic sigmoid (logistic) function was used to model signal decay as a function of peptide concentration. Additional *a priori* assumptions of the function parameters were the following:

$$p\left(k|lower=-10,upper=10\right)=\frac{1}{upper-lower}$$

$$p\left(L|lower=-3,upper=3\right)=\frac{1}{upper-lower}$$

$$p\left(b|lower=-10,upper=10\right)=\frac{1}{upper-lower}$$

The standard deviation of the observed NMR signal intensities was assumed *a priori* to be less than 5%:

$$p\left(\varepsilon |lower=0,upper=0.05\right)=\frac{1}{upper-lower}$$

The observations were modelled as normally distributed random variables with parameters:

$$p\left(I_{norm}\left(c\right)|\mu =\frac{L}{1+e^{-k\left(\log_{10}\;c-\log_{10}\;cpc\right)}}+b,\varepsilon \right)=\sqrt{\frac{1}{2\pi \varepsilon }}e^{-\frac{1}{2\varepsilon }{\left(I_{norm}\left(c\right)-\mu \right)}^{2}}$$

1000 posterior samples were collected in four parallel chains using the No-U-Turn sampling algorithm (NUTS) after 2000 tuning steps/chain.^83^ The default sampling parameters were used except for “target accept” parameter, which was increased to 0.95. For the final analysis the 1000 samples from the four chains were merged to yield 4000 samples.

#### BLI progress curve analysis

Δλ(t) values were converted to dimensionless quantities by dividing the wavelength shifts with 1 nm prior to the analysis. To develop the model, we tentatively assumed Langmuir’s 1:1 model.^85^ The kinetic modelling was performed with the pymc3 python package^66^ using a Bayesian model:

$$p\left(K_{D,app}|lower=10^{-15},upper=10^{-3}\right)=\frac{1}{upper-lower}$$

$$p\left(k_{-1}|lower=10^{-20},upper=10\right)=\frac{1}{upper-lower}$$

$$k_{1}=\frac{k_{-1}}{K_{D,app}}$$

$$p\left(A|lower=10^{-6},upper=10\right)=\frac{1}{upper-lower}$$

$$p\left(\varepsilon |lower=10^{-6},upper=1\right)=\frac{1}{upper-lower}$$

$$p\left(\Delta \lambda \left(0\right)|\mu =\left\langle {\Delta \lambda }_{i}\right\rangle ,\sigma =\frac{1}{10}\sqrt{\frac{\sum {\left({\Delta \lambda }_{i}-\left\langle {\Delta \lambda }_{i}\right\rangle \right)}^{2}}{n}}\right)=\sqrt{\frac{1}{2\pi \sigma }}e^{-\frac{1}{2\sigma }{\left(\Delta \lambda \left(t\right)-\mu \right)}^{2}};\Delta \lambda \left(0\right)\in \left[10^{-20},+\infty \right]$$

The association and dissociation phases were modelled simultaneously, and the probability of wavelength shift during association phase was modeled as:

$$p\left(\Delta \lambda \left(t\right)|\mu =\frac{Ac}{K_{D,app}+c}\left(1-e^{-\left(k_{1}c+k_{-1}\right)t}\right),\sigma =\varepsilon \right)=\sqrt{\frac{1}{2\pi \sigma }}e^{-\frac{1}{2\sigma }{\left(\Delta \lambda \left(t\right)-\mu \right)}^{2}}$$

The probability of wavelength shift during the dissociation phase was modelled as:

$$p\left(\Delta \lambda \left(t\right)|\mu =\Delta \lambda \left(0\right)e^{-k_{-1}t},\sigma =\varepsilon \right)=\sqrt{\frac{1}{2\pi \sigma }}e^{-\frac{1}{2\sigma }{\left(\Delta \lambda \left(t\right)-\mu \right)}^{2}}\text{,}$$

where *A* and *k*_*1*_ are the amplitude and rate constant of the exponential association process, respectively. The concentration of the analyte is *c* and *K*_*D,app*_ is the apparent dissociation constant. The dissociation rate constant is marked with *k*_*-1*_. ε represents the standard deviation of Δλ(t). The total amplitude of wavelength shift is the wavelength shift at the beginning of the dissociation phase (Δλ(0)). Its prior distribution is modeled by a truncated normal distribution with parameters based on an empirical Bayes estimate of the starting observations. The data points in the interval 0 to 1 s are represented by the index *i*. The residual plots from the Bayesian model used in the BLI experiment with peptide EZH2_172-211_ and survivin are shown in Figure S6.

## Data Availability

• Data including peptide microarray raw data spreadsheet, the NMR spectra, the BLI progress curves have been deposited at the Zenodo database. The DOI is listed in the key resources table. RNA sequencing data and survivin ChIP-Seq data (fastq-files and processed files) of CD4^+^ T cells that support the findings of this study have been deposited in NCBI GEO. The survivin SAXS data is deposited at the SASBDB. • All original code has been deposited at the Zenodo and Github databases and is publicly available as of the date of publication. DOIs and depositories are listed in the key resources table. • Other data that support the findings of this study are available upon reasonable request and by contacting the lead contact author.

## Supplemental information

Document S1. Figures S1–S11 and Table S2–S11

Table S1. Differentially expressed protein coding genes connected to the changeable H3K27me3 peaks in CD4^+^ T cells, related to Figure 1 CD4^+^ T cells (n=4) were cultured with survivin inhibitor YM155 (0 and 10 ng/ml). Expression was analyzed by RNA-seq and compared by paired DESeq2 test.
