# Supplementary material for: Survivin prevents the polycomb repressor complex 2 from methylating histone 3 lysine 27
Source: iScience. 2023 May 29;26(7):106976. doi: 10.1016/j.isci.2023.106976 (PMC10391610; doi:10.1016/j.isci.2023.106976)
Supplement: Document S1. Figures S1–S11 and Table S2–S11 [file mmc1.pdf]

iScience, Volume *26*

Supplemental information

Survivin prevents the polycomb repressor complex 2 from methylating histone 3 lysine 27

Maja Jensen, Venkataragavan Chandrasekaran, María-José García-Bonete, Shuxiang Li, Atsarina Larasati Anindya, Karin Andersson, Malin C. Erlandsson, Nina Y. Oparina, Björn M. Burmann, Ulrika Brath, Anna R. Panchenko, Maria Bokarewa I., and Gergely Katona

Table S2 Annotated features in the EZH2 peptides interacting with survivin, Related to Figure 2.

EZH2	Sequence	Fluorescence intensity (au. 0-64193)	Comments
S21	KSEKGPVCWRKRVKS	4969	Phosphorylation. methyltransferase activity [S1]
	PVCWRKRVKSEYMRL	679	
	KRVKSEYMRLRQLKR	19209	
P132 & Y133			Weaver syndrome. impairs methyltransferase [S2]
	VEDETVLHNIPYMGD	1640	
	VLHNIPYMGDEVLDQ	889	
	PYMGDEVLDQDGTFI	1993	
C588	PDLCLTCGAADHWDS	1287	Impairs methyltransferase [S3]
	TCGAADHWDSKNVSC	682	
Y641	VQKNEFISEYCGEII	1037	active site
	FISEYCGEIISQDEA	2238	
Y661			Binding site for pyridone-containing inhibitors [S4]
	SQDEADRRGKVYDKY	852	
	DRRGKVYDKYMCSFL	294	
Y726	AKRAIQTGEELFFDY	17722	active site
	QTGEELFFDYRYSQA	1545	
Y726 & Y736	LFFDYRYSQADALKY	957	active site & Weaver syndrome [S5]
Y736 & E740	RYSQADALKYVGIER	552	Weaver syndrome [S5]
	DALKYVGIEREMEIP	82	

Table S3 Annotated features in the EED interacting peptides, Related to Figure 2.

EED	Sequence	Fluorescence intensity (au. 0-64193)	Comments
K70-K79	NTPNAPGRKSWGKGK	2749	DNA binding [S6]
	PGRKSWGKGKWKSKK	13033	
	WGKGKWKSKKCKYSF	5143	
	WKSKKCKYSFKCVNS	112	
Y148	YVDADADENF**Y**TCAW	5116	aromatic cage [S7]
	ADENF**Y**TCAWTYDSN	1628	
R236	DTLVAIFGGVEGH**R**D	539	Weaver syndrome
	IFGGVEGH**R**DEVLSA	93	
	EGH**R**DEVLSADYDLL	5179	
R302 & Y308		579	Weaver syndrome. alanine binding pocket
	DFST**R**DIHRN**Y**VDCV		
	DIHRN**Y**VDCVRWLGD	1492	
C324	LILSKS**C**ENAIVCWK	120	alanine binding pocket [S7]
W364 & Y365	LGRFDYSQCDI**WY**MR	678	aromatic cage [S7]
	YSQCDI**WY**MRFSMDF	1624	

Table S4 Annotated features in the SUZ12 interacting peptides, Related to Figure 2.

SUZ12	Sequence	Fluorescence intensity (au. 0-64193)	Comments
Zinc finger (448 – 471)	EARDDLHCPWCTLNC	1389	
	CTLNCRKLYSLLKHL	338	
	LLKHLKLCHSRFIFN	212	
E610	TQIEEFSDVNEGEKE	2471	Weaver syndrome [S8]
	FSDVNEGEKEVMKLW	646	

Table S5 Annotated features in the JARID2 interacting peptides, Related to Figure 2.

JARID2	Sequence	Fluorescence intensity (au. 0-64193)
ARID domain	RWGPNVQRLACIKKH	3046
	VQRLACIKKHLKSQG	165
	LKSQGITMDELPLIG	56
	ITMDELPLIGGCELD	6829
	LPLIGGCELDLACFF	2771
	GCELDLACFFRLINE	1943
	AQDRLAKLQEAYCQY	730
	AKLQEAYCQYLLSYD	7908
	AYCQYLLSYDSLSPE	1935
	LLSYDSLSPEEHRRL	316

Table S6 Amino acid – atom type conversion table used for peptide analysis, Related to STAR Methods.

aa	CA-Gly	Pro-MC	Carboxyl	Amide	His	Trp	Phe-Tyr	OH-Tyr	CH2	CH	CH3	OH	SH	S	NH3	Arg	MC
**A**	0	0	0	0	0	0	0	0	0	0	1	0	0	0	0	0	4
**C**	0	0	0	0	0	0	0	0	1	0	0	0	1	0	0	0	4
**D**	0	0	3	0	0	0	0	0	1	0	0	0	0	0	0	0	4
**E**	0	0	3	0	0	0	0	0	2	0	0	0	0	0	0	0	4
**F**	0	0	0	0	0	0	6	0	1	0	0	0	0	0	0	0	4
**G**	1	0	0	0	0	0	0	0	0	0	0	0	0	0	0	0	3
**H**	0	0	0	0	5	0	0	0	1	0	0	0	0	0	0	0	4
**I**	0	0	0	0	0	0	0	0	1	1	2	0	0	0	0	0	4
**K**	0	0	0	0	0	0	0	0	4	0	0	0	0	0	1	0	4
**L**	0	0	0	0	0	0	0	0	1	1	2	0	0	0	0	0	4
**M**	0	0	0	0	0	0	0	0	2	0	1	0	0	1	0	0	4
**N**	0	0	0	3	0	0	0	0	1	0	0	0	0	0	0	0	4
**P**	0	2	0	0	0	0	0	0	3	0	0	0	0	0	0	0	2
**Q**	0	0	0	3	0	0	0	0	2	0	0	0	0	0	0	0	4
**R**	0	0	0	0	0	0	0	0	3	0	0	0	0	0	0	4	4
**S**	0	0	0	0	0	0	0	0	1	0	0	1	0	0	0	0	4
**T**	0	0	0	0	0	0	0	0	0	1	1	1	0	0	0	0	4
**Y**	0	0	0	0	0	0	6	1	1	0	0	0	0	0	0	0	4
**V**	0	0	0	0	0	0	0	0	0	1	2	0	0	0	0	0	4
**W**	0	0	0	0	0	9	0	0	1	0	0	0	0	0	0	0	4

Table S7 SAXS data collection. processing and modeling statistics, Related to Star Methods.

**Data collection parameters**			
Beamline	ESRF BM29, Grenoble, France		
Beam geometry (µm^2)	700 x 700		
Wavelength (nm)	0.099		
Detector	Pilatus 1M in vacuum		
Detector distance (m)	2.867		
q range (nm^{-1})	0.03 – 5		
Sample environment	Quartz glass capillary. 1mm diameter		
Exposure time (s) per frame	1		
Temperature (°C)	10		
Mode	SEC-SAXS		
**SEC-SAXS parameters**			
Column	Superdex 75 100/300GL		
Injected volume (µl)	500		
Concentration (mg/ml)	≈ 3.0		
Flow rate (ml/min)	0.6		
Solvent composition	50 mM Tris pH8, 150 mM NaCl, 1 mM DTT		
Frames	**Total**	**Buffer**	**Protein**
	2500	293	33
**Structural parameters**			
Guinier analysis			
I(0) (cm^{-1})	8.36 ± 0.026		
R^g (nm)	2.88 ± 0.02		
q R_g-range (nm^{-1})	0.01-045		
Total quality estimate (AutoRg)[S9]	0.99		
P(r) analysis			
I(0) (cm^{-1})	8.39		
R_g (nm)	2.94		
q-range (nm^{-1})	0.01-2.77		
D_{max} (nm)	10		
Total quality estimate (GNOM) [S10]	0.67		
Porod volume estimate. V_p (nm^3)	53.15		
**Molecular weight determination**			
Theoretical MW from the sequence	16.80 kDa (monomer)		
From Qp	32.69		
From MoW (kDa) [S11]	33.18		
From V_c (kDa) [S12]	33.77		
Volume of correlation (V_c)	346		
From Size & Shape (kDa) [S13]	36.92		
From Bayesian inference			
Credibility Interval (kDa)	31.3 – 34.95		
Credibility Interval Probability (%)	95.63		
**Atomic modelling**			
Model	6SHO [S14]		
CRYSOL			
χ^2	1.29		
**Software**			
SAXS data reduction	BsxCuBE / CHROMIXS [S15]		
Data processing	Primusqt - ATSAS package (v.3.0.0) [S16,17]		
Ab initio model	DAMMIF[S13]		
Model validation. averaging and final refinement	DAMMIN[S18], DAMAVER [S19]		
Calculation of theoretical intensity and comparison with SAXS data	CRYSOL[S20]		
3D graphics representations	UCSF ChimeraX (v1.2.5) [S21]		
SASBDB entry	SASDR86		

Table S8 The posterior distribution of the global parameters used to model the survivin self-titration microscale thermophoresis experiment, Related to Figure 6.

	mean	sd	$hdi_{3\%}$	$hdi_{97\%}$
K_d (nM)	1406	357	741	2050
B (F_{norm})	0.148	0.002	0.144	0.152
U (F_{norm})	0.056	0.004	0.048	0.063
ε (F_{norm})	0.001	0.000	0.001	0.002

Table S9 Posterior distribution of model parameters used to model the BLI progress curves, Related to Figure 5. The mean and standard deviation of the posterior samples are reported.

Exp.	Conc.	A (nm)	$K_{D,app}$ (M)	$\Delta\lambda(0)$ (nm)	k_{-1} (s^{-1})	k_1 ($M^{-1}s^{-1}$)	ε (nm)
$EZH2_{172-211}$	100 nM	0.23 ± 1.1×10^{-4}	7.0×10^{-10} ± 6.7×10^{-12}	2.3×10^{-1} ± 7.5×10^{-5}	2.3×10^{-1} ± 7.5×10^{-5}	5.0×10^{4} ± 84	3.5×10^{-3} ± 2.0×10^{-5}
$EZH2_{172-211}$	33 nM	0.11 ± 1.8×10^{-4}	3.5×10^{-10} ± 6.5×10^{-12}	1.0×10^{-1} ± 5.9×10^{-5}	1.0×10^{-1} ± 5.9×10^{-5}	8.8×10^{4} ± 3.3×10^{2}	2.9×10^{-3} ± 1.7×10^{-5}
$EZH2_{172-211}$	11 nM	5.5×10^{-2} ± 5.5×10^{-4}	6.1×10^{-10} ± 1.4×10^{-11}	4.0×10^{-2} ± 4.5×10^{-5}	4.0×10^{-2} ± 4.5×10^{-5}	1.3×10^{5} ± 2.2×10^{3}	2.9×10^{-3} ± 1.8×10^{-5}
$EZH2_{172-211}$	3.7 nM	0.18 ± 0.21	6.7×10^{-4} ± 2.4×10^{-4}	3.9×10^{-7} ± 3.8×10^{-7}	3.9×10^{-7} ± 3.8×10^{-7}	1.1×10^{4} ± 2.5×10^{4}	4.3×10^{-3} ± 2.7×10^{-5}
$EZH2_{172-211}$ (reverse)	21.2 µM	0.13 ± 1.2×10^{-3}	1.9×10^{-9} ± 1.3×10^{-9}	7.0×10^{-2} ± 3.1×10^{-5}	7.0×10^{-2} ± 3.1×10^{-5}	45 ± 0.59	3.0×10^{-3} ± 1.9×10^{-5}
$EZH2_{172-211}$ (reverse)	2.12 µM	3.6×10^{-2} ± 1.8×10^{-4}	3.1×10^{-11} ± 2.1×10^{-11}	3.9×10^{-2} ± 3.7×10^{-5}	3.9×10^{-2} ± 3.7×10^{-5}	1.6×10^{3} ± 20	3.8×10^{-3} ± 2.3×10^{-5}
$EZH2_{172-211}$ (reverse)	212 nM	2.1×10^{-2} ± 7.2×10^{-4}	1.0×10^{-11} ± 6.7×10^{-12}	2.4×10^{-2} ± 4.5×10^{-5}	2.4×10^{-2} ± 4.5×10^{-5}	7.5×10^{3} ± 4.2×10^{2}	4.3×10^{-3} ± 2.7×10^{-5}
$EZH2_{172-211}$ (reverse)	21.2 nM	8.0×10^{-3} ± 3.6×10^{-4}	2.8×10^{-12} ± 2.1×10^{-12}	9.7×10^{-3} ± 3.4×10^{-5}	9.7×10^{-3} ± 3.4×10^{-5}	9.0×10^{4} ± 7.2×10^{3}	3.3×10^{-3} ± 2.0×10^{-5}

Table S10 Posterior distribution of the model parameters used to model the NMR signal decay, Related to Figure 6. The mean and highest density interval (95%) of the posterior samples are indicated.

Peptide	EZH2$_{172-211}$	EED$_{128-152}$	SUZ12$_{573-597}$	JARID2$_{170-194}$
**<cpc>**	2.1×10^{-3}	7.6×10^{-3}	6.6×10^{-3}	8.5×10^{-3}
**cpc HDI95%**	2.7×10^{-4} - 3.4×10^{-3}	5.8×10^{-3} - 9.7×10^{-3}	2.1×10^{-3} - 9.4×10^{-3}	6.1×10^{-3} - 1.1×10^{-2}
**<L>**	0.68	-0.00	-0.59	-0.53
**L HDI95%**	-1.65 - 2.11	-1.17 - 1.14	-1.68 - 1.39	-1.21 - 1.13
**<k>**	-1.04	-0.01	0.99	1.25
**k HDI95%**	-2.90 - 2.54	-4.46 - 4.52	-2.50 - 2.59	-3.15 - 3.62
**<ε>**	0.03	0.04	0.03	0.03
**ε HDI95%**	0.02 - 0.05	0.02 - 0.05	0.01 - 0.05	0.01 - 0.05
**<b>**	0.32	0.51	0.87	0.79
**b HDI95%**	-0.11 - 1.42	-0.07 - 1.12	-0.09 - 1.45	-0.06 - 1.16

Table S11 Docking energy scores of the top 10 docking conformations, Related to STAR Methods.

Docking conformations	Docking energy scores (kcal/mol)
1	-345.66
2	-331.72
3	-321.41
4	-315.61
5	-307.39
6	-307.13
7	-302.36
8	-297.28
9	-296.17
10	-289.60

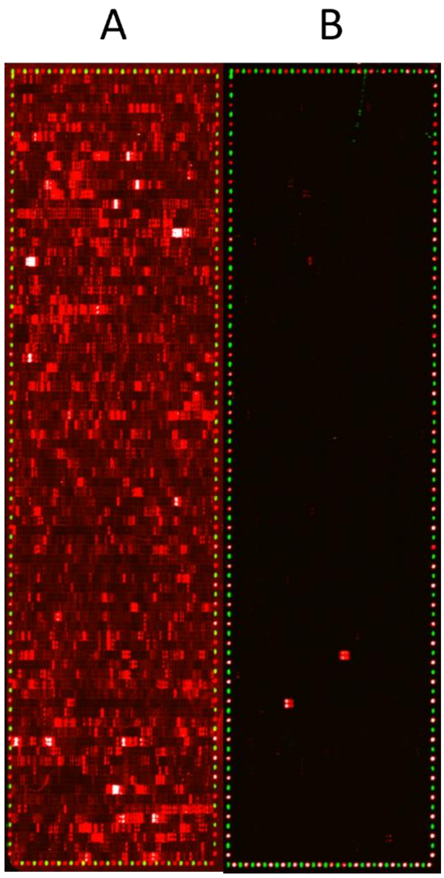

Figure S1 Raw fluorescence scans of the peptide microarray, Related to Figures 2 and 3. (**A**) incubated with 1 µg/mL survivin and labelled anti-His tag antibody (**B**) only labelled anti-His tag antibody.

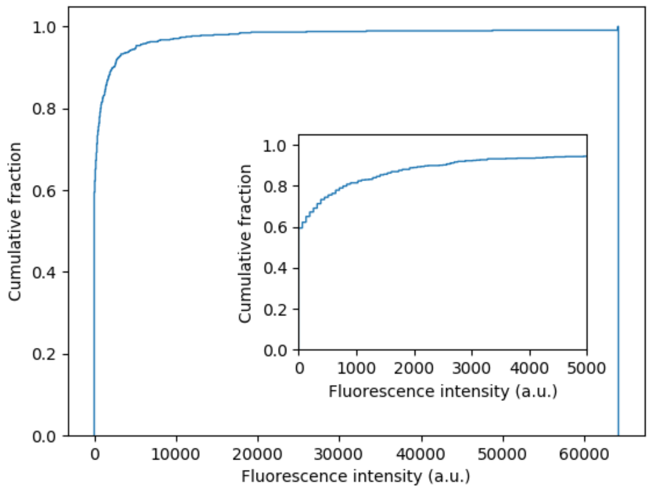

Figure S2 The cumulative fraction of peptides with fluorescence intensities less than a certain threshold in the peptide microarray experiment, Related to Figure 4.

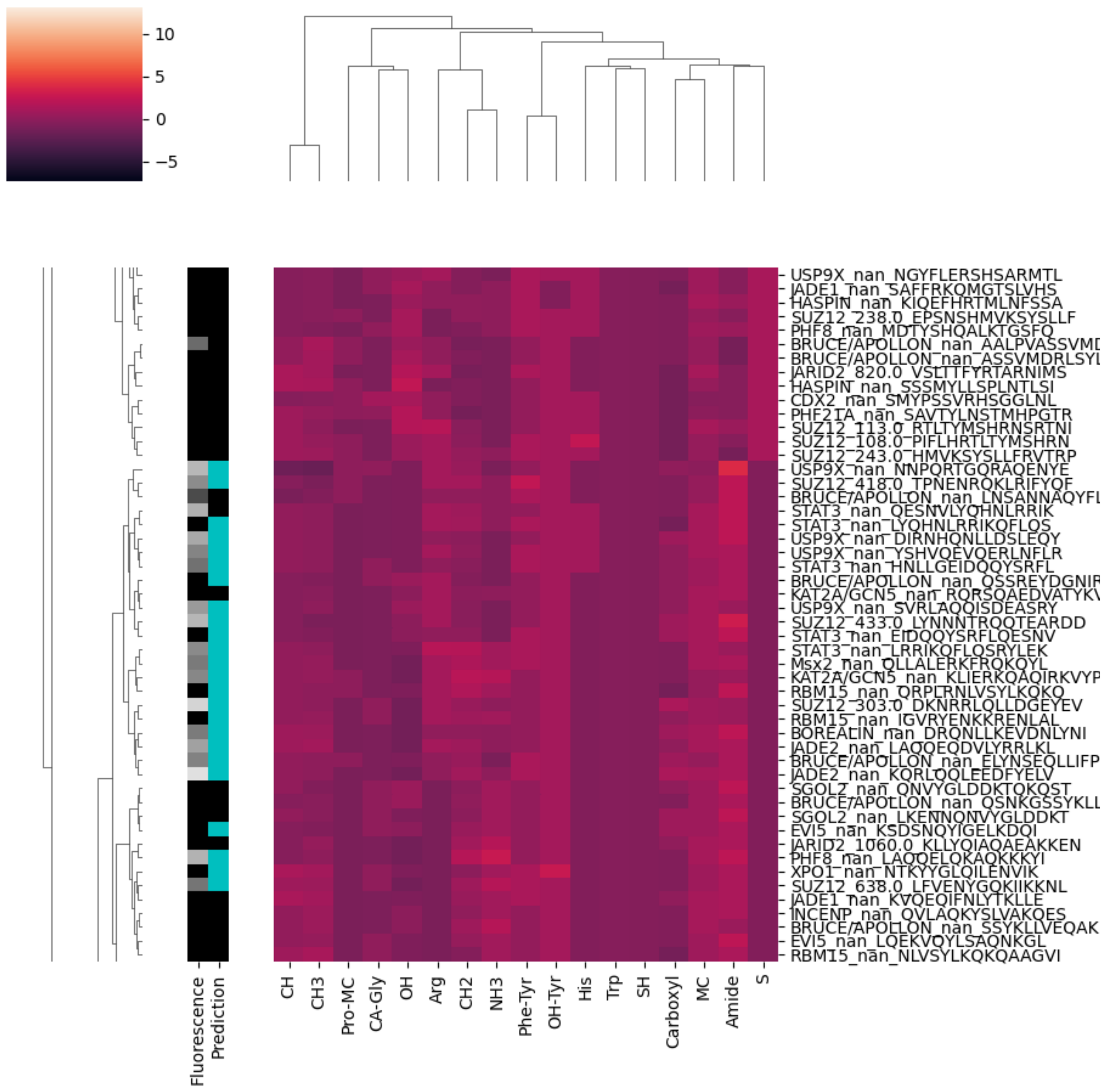

Figure S3 A detailed look at the relationship between survivin interaction with peptides and peptide composition, Related to Figure 4. A magnified view of the cluster, revealing individual peptides. The heat map depicts the light/dark high/low abundance of atom types (z-score). The grayscale bar represents the logarithm of the peptide's fluorescence intensity in the survivin peptide microarray experiment (*black* zero intensity. *white* highest level of intensity). The prediction bar indicates the accuracy of the machine learning prediction using atom type abundance as features (*black* and *cyan* colors mark predicted non-interacting and interacting peptides, respectively).

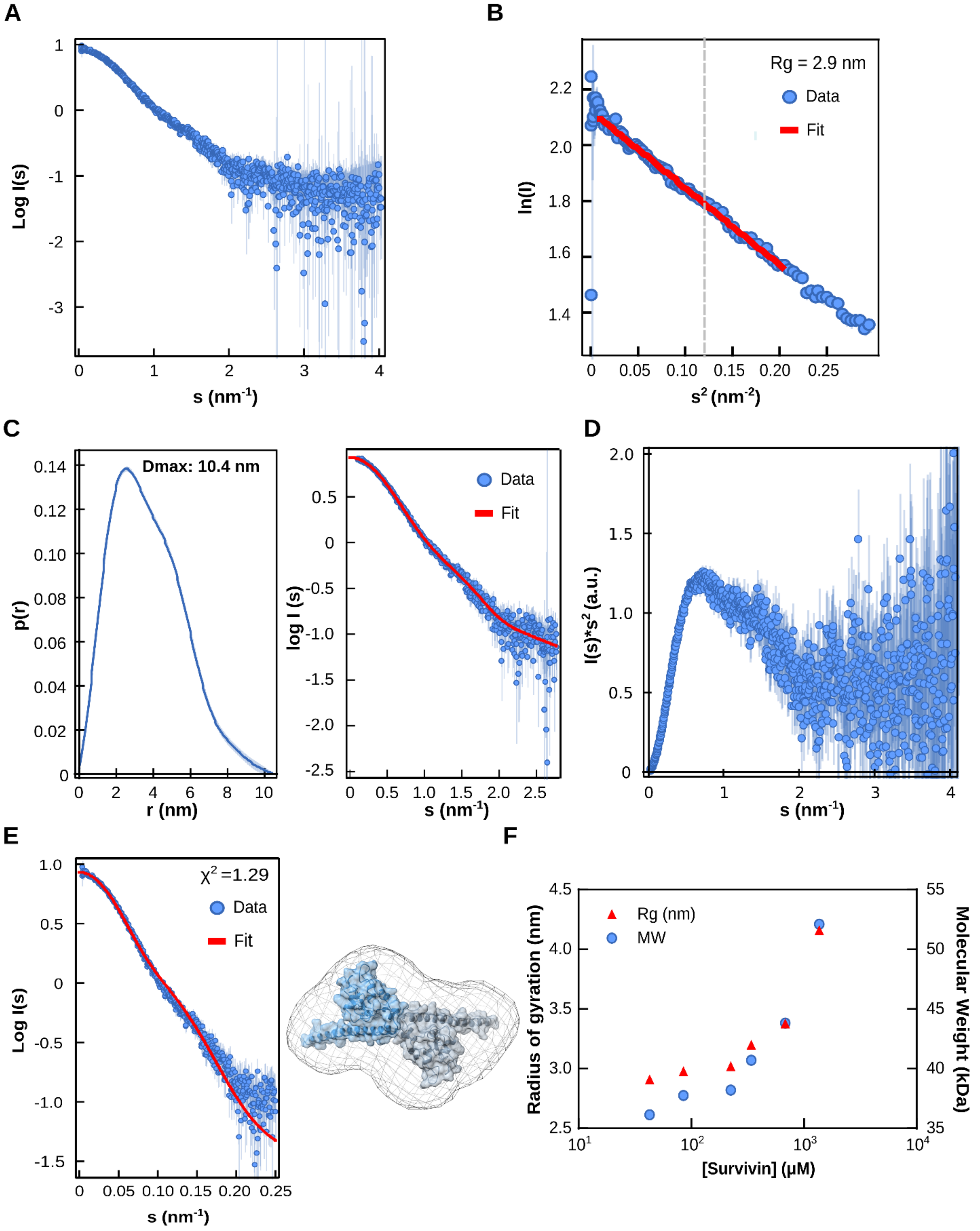

Figure S4 SAXS experiment of survivin solution, Related to Figure 6. (A) Survivin SAXS scattering curve. (B) Guinier-transform and plotting of the scattering contrast data shows linear relationship. (C) Asymmetric p(r) function and its Fourier transform. (D) Kratky plot of survivin SAXS data (E) Crysol[S20] modelling of the SAXS curve based on the dimeric survivin structure in the PDB entry 6SHO.[S14] DAMAVER *ab initio* model superposed to the 6SHO crystal structure. (F) Radius of gyration (nm) and molecular weight estimation (kDa) as a function of survivin concentration in SAXS batch experiments.

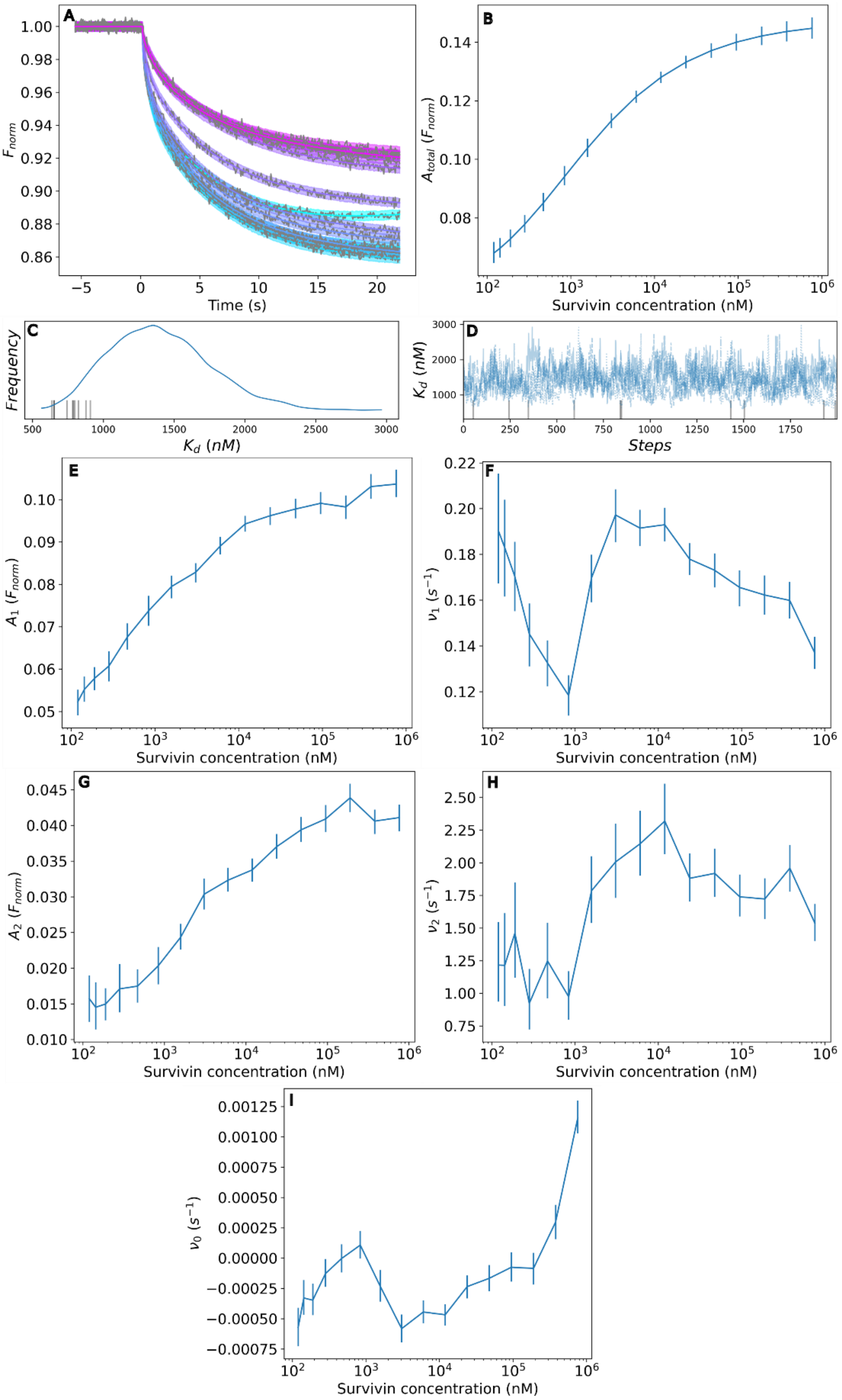

Figure S5 Survivin self-titration monitored by microscale thermophoresis, Related to Figure 6. (A) Raw progress curves (*gray*) overlayed with the posterior predictions ranges and mean predictions. The magenta-cyan color scale is advancing with increasing survivin concentration. (B) Posterior distributions of $A_{total,n}$ (C) Posterior distribution and (D) samples of the K_d parameter. (E) Posterior distribution of $A_{1,n}$ parameters. (F) Posterior distribution of $v_{1,n}$ parameters. (G) Posterior distribution of $A_{2,n}$ parameters. (H) Posterior distribution of $v_{2,n}$ parameters. (I) Posterior distribution of $v_{0,n}$ parameters.

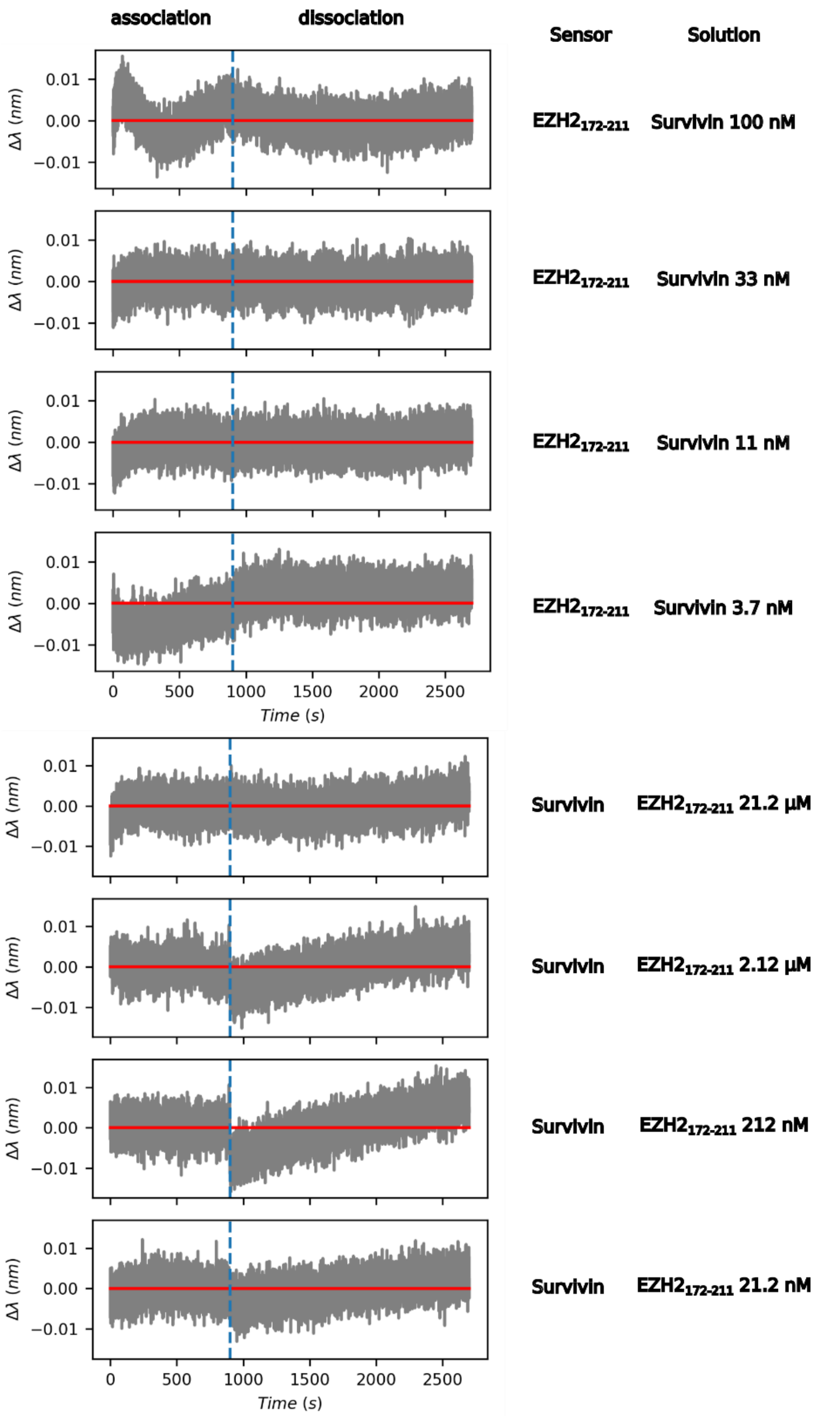

Figure S6 Residual plots of the combined association and dissociation phases of the BLI experiment with peptide EZH2$_{172\text{-}211}$ and survivin in two configurations (EZH2$_{172\text{-}211}$ peptide bound to the sensor with survivin in solution and the reverse configuration), Related to Figure 5. Each row represents one experimental data set. The residuals correspond to the 1:1 model that was used for both phases of the experiment (association and dissociation). The association and dissociation phases are separated by blue vertical dashed lines.

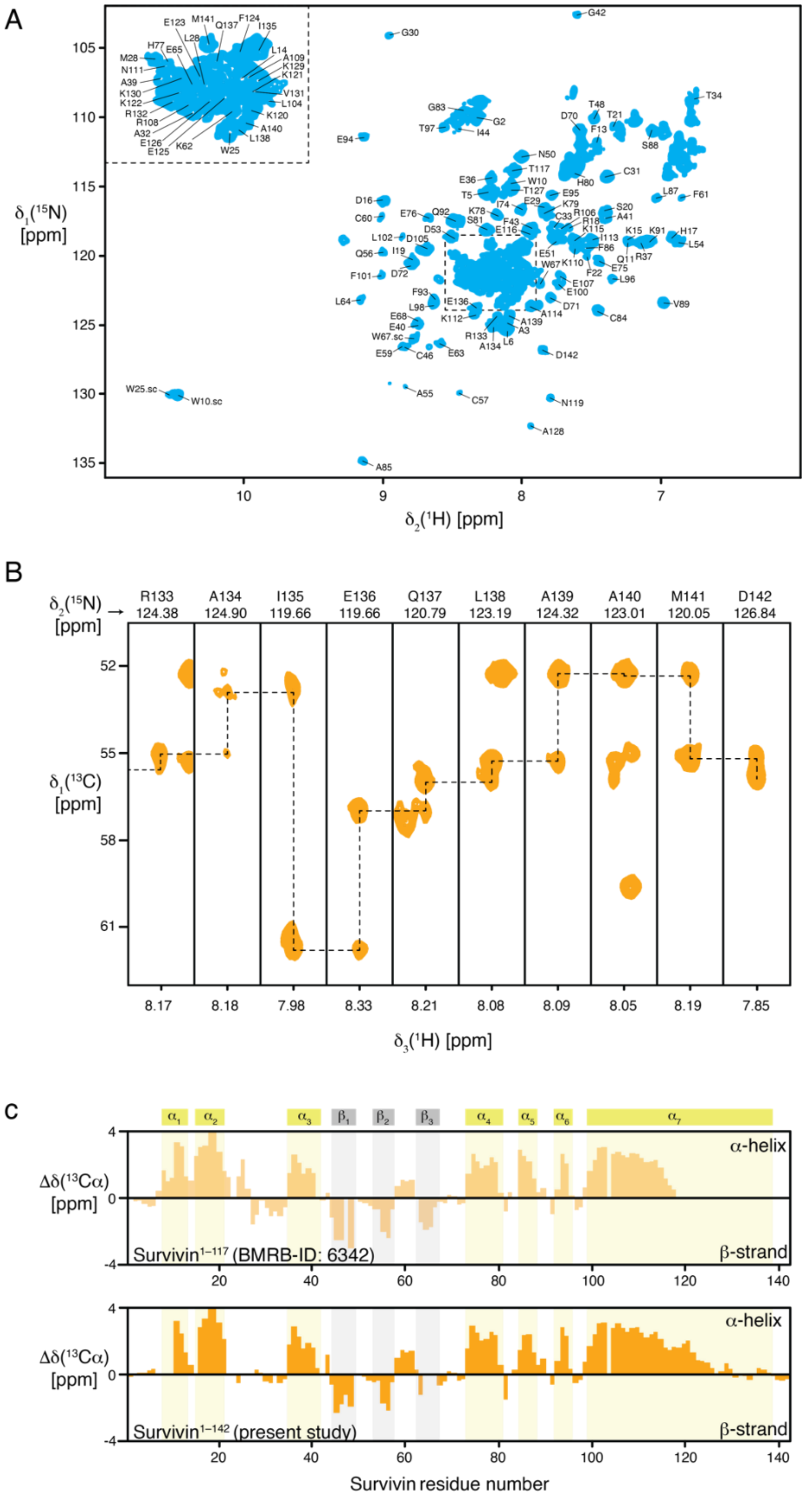

Figure S7 Sequence specific NMR resonance assignment of survivin$^{1\text{-}142}$, Related to Figure 6. (A) 2D [^{15}N,^{1}H]-NMR spectrum of survivin$^{1\text{-}142}$. The sequence-specific resonance assignment obtained as outlined in the methods section are annotated. The central part is enlarged in the upper right corner. “sc” refers to the tryptophane indole resonances. (B) Strips of residues 133–142 from a 3D HNCA experiment. The broken line indicates the sequential connection between the strips for ^{13}Cα. (C) Secondary ^{13}C chemical shifts of the ^{13}Cα plotted against the survivin residue number. Secondary structure elements were calculated based on the survivin crystal structure (PDB ID: 3UEF) are indicated by the lemon or grey bars on top and the respective shading in the plots. Plots show the secondary ^{13}Cα chemical shifts of the survivin$^{1\text{-}117}$(top), determined previously (BMRB ID: 6342), as well as the corresponding data for survivin$^{1\text{-}142}$ (bottom), determined in the present study.

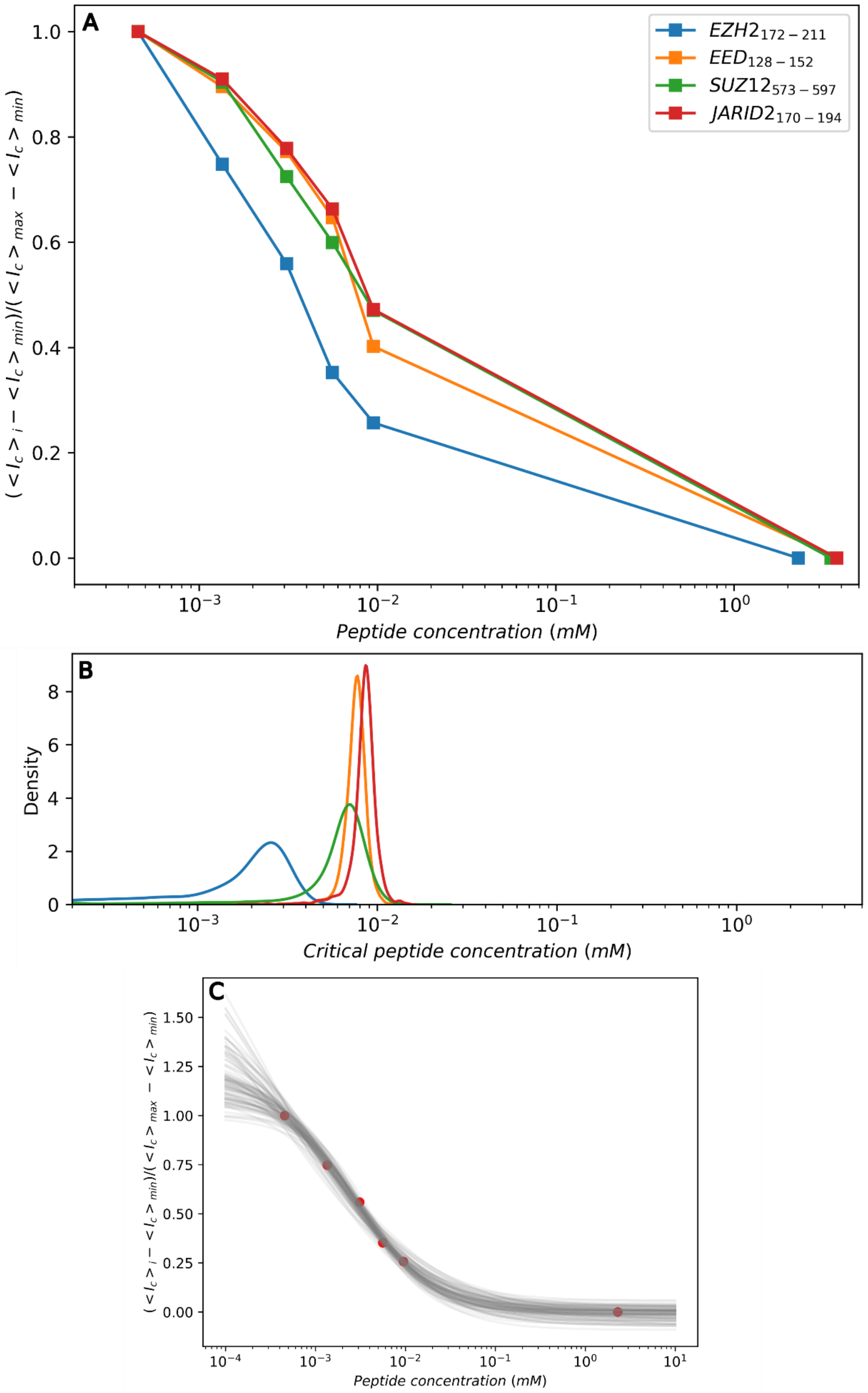

Figure S8 Global analysis of the NMR signal, Related to Figure 6. (A) The loss of the survivin NMR spectral signal as a function of peptide concentration. (B) The estimated posterior distribution of critical peptide concentration was used to predict NMR signal attenuation. (C) Posterior predictions of the logistic function (100 *gray* lines) and survivin NMR signal intensity (*red*) when titrating with the peptide $EZH2_{172-211}$.

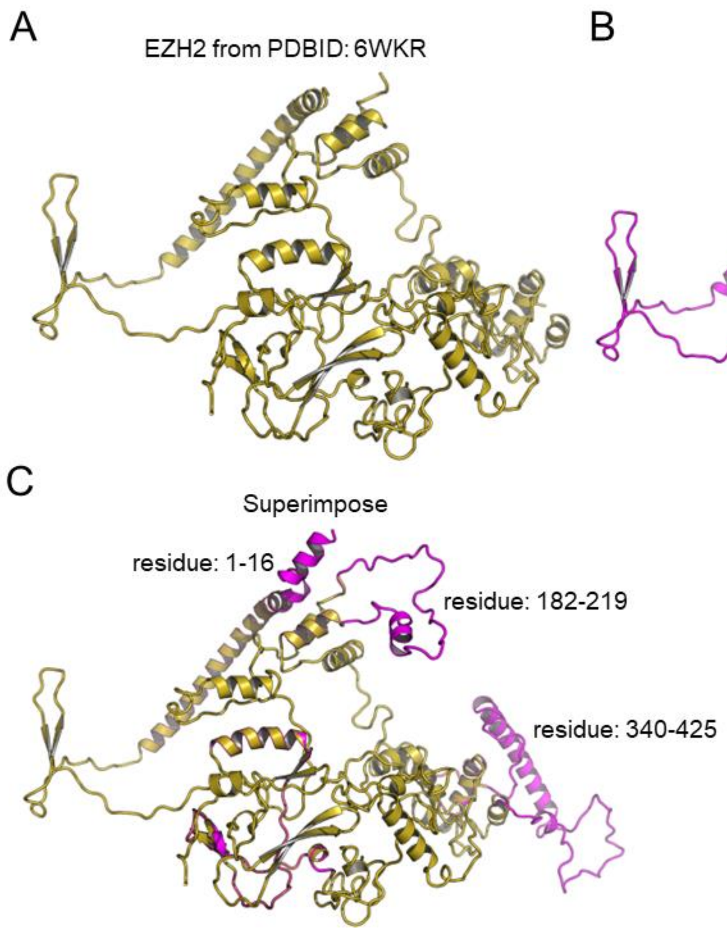

Figure S9 Molecular modeling of the EZH2 subunit, Related to Figure 7. (A) The cryo-EM structure of the EZH2 subunit from the PRC2 complex (PDB ID: 6WKR) [S22]. (B) The modelled structure of the EZH2 subunit. (C) Structural superimposition of the modelled and cryo-EM structure of EZH2.

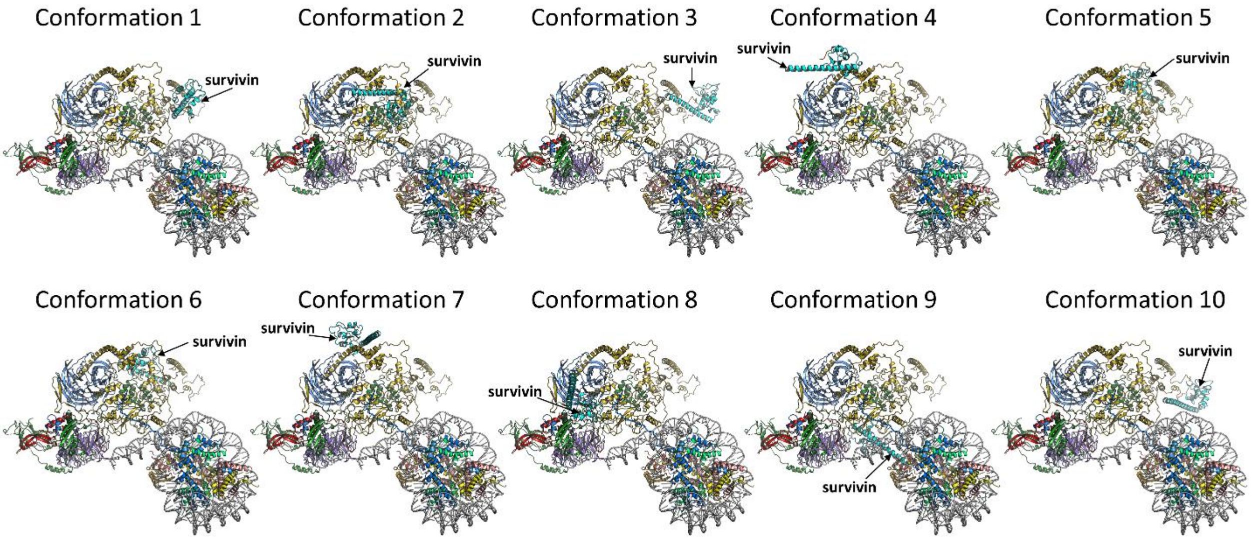

Figure S10 The top 10 molecular docking conformations of survivin binding with the PCR2 complex, Related to Figure 7.

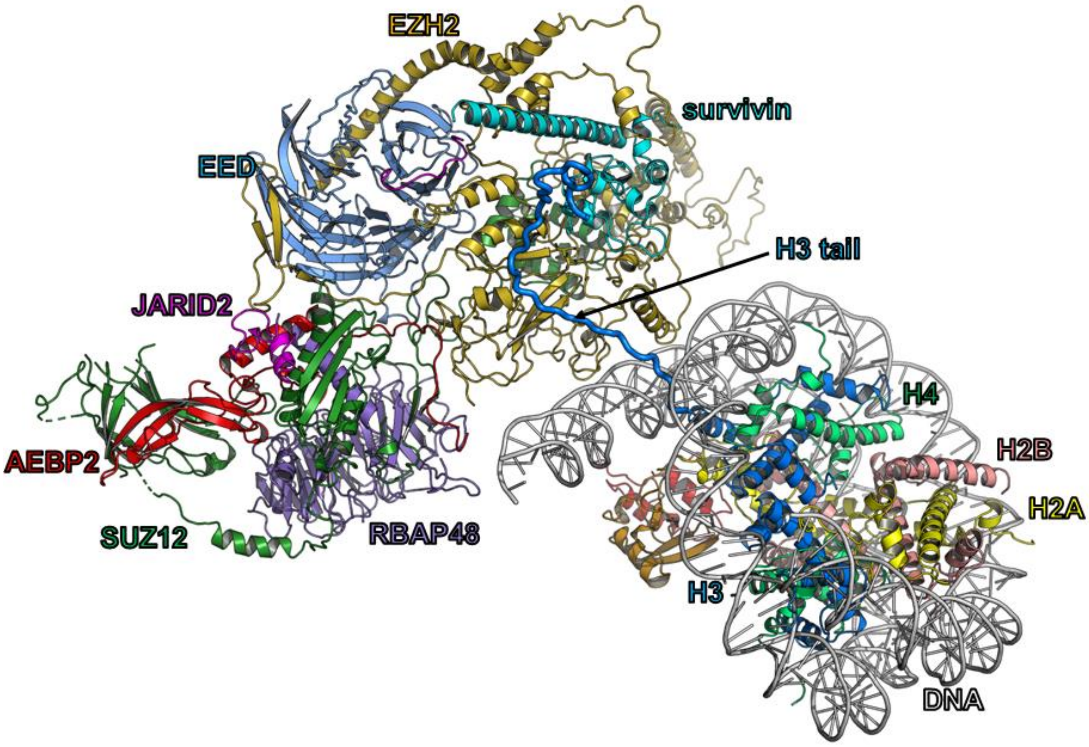

Figure S11 The final structural model of survivin binding with the PRC2 complex, Related to Figure 7.

References

S1. Cha, T.L., Zhou, B.P., Xia, W., Wu, Y., Yang, C.C., Chen, C.T., Ping, B., Otte, A.P., and Hung, M.C. (2005). Akt-mediated phosphorylation of EZH2 suppresses methylation of lysine 27 in histone H3. Science *310*, 306-310. 10.1126/science.1118947.

S2. Cohen, A.S.A., Yap, D.B., Lewis, M.E.S., Chijiwa, C., Ramos-Arroyo, M.A., Tkachenko, N., Milano, V., Fradin, M., McKinnon, M.L., Townsend, K.N., et al. (2016). Weaver Syndrome-Associated EZH2 Protein Variants Show Impaired Histone Methyltransferase Function In Vitro. Hum Mutat *37*, 301-307. 10.1002/humu.22946.

S3. Kuzmichev, A., Nishioka, K., Erdjument-Bromage, H., Tempst, P., and Reinberg, D. (2002). Histone methyltransferase activity associated with a human multiprotein complex containing the Enhancer of Zeste protein. Genes Dev *16*, 2893-2905. 10.1101/gad.1035902.

S4. Bratkowski, M., Yang, X., and Liu, X. (2018). An Evolutionarily Conserved Structural Platform for PRC2 Inhibition by a Class of Ezh2 Inhibitors. Sci Rep *8*, 9092. 10.1038/s41598-018-27175-w.

S5. Tatton-Brown, K., Hanks, S., Ruark, E., Zachariou, A., Duarte Sdel, V., Ramsay, E., Snape, K., Murray, A., Perdeaux, E.R., Seal, S., et al. (2011). Germline mutations in the oncogene EZH2 cause Weaver syndrome and increased human height. Oncotarget *2*, 1127-1133. 10.18632/oncotarget.385.

S6. Poepsel, S., Kasinath, V., and Nogales, E. (2018). Cryo-EM structures of PRC2 simultaneously engaged with two functionally distinct nucleosomes. Nat. Struct. Mol. Biol. *25*, 154-162. 10.1038/s41594-018-0023-y.

S7. Margueron, R., Justin, N., Ohno, K., Sharpe, M.L., Son, J., Drury, W.J., 3rd, Voigt, P., Martin, S.R., Taylor, W.R., De Marco, V., et al. (2009). Role of the polycomb protein EED in the propagation of repressive histone marks. Nature *461*, 762-767. 10.1038/nature08398.

S8. Imagawa, E., Higashimoto, K., Sakai, Y., Numakura, C., Okamoto, N., Matsunaga, S., Ryo, A., Sato, Y., Sanefuji, M., Ihara, K., et al. (2017). Mutations in genes encoding polycomb repressive complex 2 subunits cause Weaver syndrome. Hum Mutat *38*, 637-648. 10.1002/humu.23200.

S9. Petoukhov, M.V., Konarev, P.V., Kikhney, A.G., and Svergun, D.I. (2007). ATSAS 2.1 - towards automated and web-supported small-angle scattering data analysis. J. Appl. Crystallogr. *40*, S223-S228. 10.1107/S0021889807002853.

S10. Svergun, D.I. (1992). Determination of the Regularization Parameter in Indirect-Transform Methods Using Perceptual Criteria. J. Appl. Crystallogr. *25*, 495-503. Doi 10.1107/S0021889892001663.

S11. Fischer, H., Neto, M.D., Napolitano, H.B., Polikarpov, I., and Craievich, A.F. (2010). Determination of the molecular weight of proteins in solution from a single small-angle X-ray scattering measurement on a relative scale. J. Appl. Crystallogr. *43*, 101-109. 10.1107/S0021889809043076.

S12. Rambo, R.P., and Tainer, J.A. (2013). Accurate assessment of mass, models and resolution by small-angle scattering. Nature *496*, 477-+. 10.1038/nature12070.

S13. Franke, D., and Svergun, D.I. (2009). DAMMIF, a program for rapid ab-initio shape determination in small-angle scattering. J. Appl. Crystallogr. *42*, 342-346. 10.1107/S0021889809000338.

S14. Garcia-Bonete, M.J., and Katona, G. (2019). Bayesian machine learning improves single-wavelength anomalous diffraction phasing. Acta Cryst A *75*, 851-860. 10.1107/S2053273319011446.

S15. Panjkovich, A., and Svergun, D.I. (2018). CHROMIXS: automatic and interactive analysis of chromatography-coupled small-angle X-ray scattering data. Bioinformatics *34*, 1944-1946. 10.1093/bioinformatics/btx846.

S16. Konarev, P.V., Volkov, V.V., Sokolova, A.V., Koch, M.H.J., and Svergun, D.I. (2003). PRIMUS: a Windows PC-based system for small-angle scattering data analysis. J. Appl. Crystallogr. *36*, 1277-1282. Doi 10.1107/S0021889803012779.

S17. Manalastas-Cantos, K., Konarev, P.V., Hajizadeh, N.R., Kikhney, A.G., Petoukhov, M.V., Molodenskiy, D.S., Panjkovich, A., Mertens, H.D.T., Gruzinov, A., Borges, C., et al. (2021). ATSAS 3.0: expanded functionality and new tools for small-angle scattering data analysis. J. Appl. Crystallogr. *54*, 343-355. 10.1107/S1600576720013412.

S18. Svergun, D.I. (1999). Restoring low resolution structure of biological macromolecules from solution scattering using simulated annealing. Biophys. J. *76*, 2879-2886. 10.1016/S0006-3495(99)77443-6.

S19. Volkov, V.V., and Svergun, D.I. (2003). Uniqueness of ab initio shape determination in small-angle scattering. J. Appl. Crystallogr. *36*, 860-864. 10.1107/S0021889803000268.

S20. Svergun, D., Barberato, C., and Koch, M.H.J. (1995). CRYSOL - A program to evaluate x-ray solution scattering of biological macromolecules from atomic coordinates. J. Appl. Crystallogr. *28*, 768-773. Doi 10.1107/S0021889895007047.

S21. Pettersen, E.F., Goddard, T.D., Huang, C.R.C., Meng, E.E.C., Couch, G.S., Croll, T.I., Morris, J.H., and Ferrin, T.E. (2021). UCSF ChimeraX: Structure visualization for researchers, educators, and developers. Protein Science *30*, 70-82. 10.1002/pro.3943.

S22. Kasinath, V., Beck, C., Sauer, P., Poepsel, S., Kosmatka, J., Faini, M., Toso, D., Aebersold, R., and Nogales, E. (2021). JARID2 and AEBP2 regulate PRC2 in the presence of H2AK119ub1 and other histone modifications. Science *371*. 10.1126/science.abc3393.
